# The pathogenesis of mesothelioma is driven by a dysregulated translatome

**DOI:** 10.1038/s41467-021-25173-7

**Published:** 2021-08-13

**Authors:** Stefano Grosso, Alberto Marini, Katarina Gyuraszova, Johan Vande Voorde, Aristeidis Sfakianos, Gavin D. Garland, Angela Rubio Tenor, Ryan Mordue, Tanya Chernova, Nobu Morone, Marco Sereno, Claire P. Smith, Leah Officer, Pooyeh Farahmand, Claire Rooney, David Sumpton, Madhumita Das, Ana Teodósio, Catherine Ficken, Maria Guerra Martin, Ruth V. Spriggs, Xiao-Ming Sun, Martin Bushell, Owen J. Sansom, Daniel Murphy, Marion MacFarlane, John P. C. Le Quesne, Anne E. Willis

**Affiliations:** 1 MRC Toxicology Unit, Gleeson Building, University of Cambridge, Cambridge, UK; 2 Institute of Cancer Sciences, University of Glasgow, Glasgow, UK; 3 Cancer Research UK Beatson Institute, Garscube Estate, Bearsden, UK; 4 Leicester Cancer Research Centre, University of Leicester, Leicester, UK; 5 Glenfield Hospital, Groby Road, University Hospitals Leicester NHS Trust Leicester, Leicester, UK

**Keywords:** Mesothelioma, Mechanisms of disease

## Abstract

Malignant mesothelioma (MpM) is an aggressive, invariably fatal tumour that is causally linked with asbestos exposure. The disease primarily results from loss of tumour suppressor gene function and there are no ‘druggable’ driver oncogenes associated with MpM. To identify opportunities for management of this disease we have carried out polysome profiling to define the MpM translatome. We show that in MpM there is a selective increase in the translation of mRNAs encoding proteins required for ribosome assembly and mitochondrial biogenesis. This results in an enhanced rate of mRNA translation, abnormal mitochondrial morphology and oxygen consumption, and a reprogramming of metabolic outputs. These alterations delimit the cellular capacity for protein biosynthesis, accelerate growth and drive disease progression. Importantly, we show that inhibition of mRNA translation, particularly through combined pharmacological targeting of mTORC1 and 2, reverses these changes and inhibits malignant cell growth in vitro and in ex-vivo tumour tissue from patients with end-stage disease. Critically, we show that these pharmacological interventions prolong survival in animal models of asbestos-induced mesothelioma, providing the basis for a targeted, viable therapeutic option for patients with this incurable disease.

## Abstract (web summary)

Treating malignant pleural mesothelioma (MpM) is challenging due to a lack of druggable genes, but other molecular features could be clinically useful. Here the authors profile mRNA translation dysregulation in MpM cell lines using polysome profiling, and identify mTORC1 and 2 as potential pharmacological targets.

## Introduction

Malignant pleural Mesothelioma (MpM) is an aggressive, near-universally fatal malignancy of the pleura that is almost exclusively associated with exposure to asbestos^1–4^. There is no effective treatment and, after a long latency period following initial exposure, it progresses rapidly^5,6^. At present, the standard of care for MpM is chemotherapy based on a combination of pemetrexed and cisplatin or radical surgery^6^, both of which are at best modestly effective at prolonging the life of patients, although emerging therapies, which target immune system checkpoints may be of value in the future^7,8^.

The mechanisms of carcinogenesis driven by asbestos exposure are poorly understood and our limited knowledge of the molecular changes associated with MpM severely hampers therapeutic development^5^. Extensive genomic analyses have been performed on MpM tumours^9–12^ and for the most part, these were found to be enriched for mutations and/or other genetic alterations (e.g., splicing changes and gene deletions) that result in a decrease in tumour suppressor gene function rather than the acquisition of mutations that activate proto-oncogenes^10,11^. For example, several human studies have shown that *p16INK4a, p14ARF, NF2* and *BAP1* are altered in MpM, with *p16INK4a* and *p14ARF* deleted in 80–90% of cases^11,13–15^ and *BAP1*^16^ and *NF2*^14^, which are mutated/deleted in over 20% of cases^11,17^. The central role of these genes in the development of MpM is corroborated by animal studies showing that their deletion/inactivation results in the greatly accelerated development of aggressive mesotheliomas in asbestos-exposed animals^17–20^. Moreover, mouse studies have also shown that hypermethylation of *p16/Ink4a* and *p19/Arf* in asbestos-induced inflammatory lesions precedes mesothelioma, resulting in silencing of *cdkn2A (Ink4a/Arf*) and loss of p16 and p19 protein^20^.

In addition to mutations and deletions in DNA that initiate tumorigenesis, it is well accepted that dysregulated cytoplasmic control of gene expression at the level of mRNA translation has a major role in both cancer development and progression^21,22^. In general, global translation initiation rates are regulated by ternary complex recycling and eukaryotic initiation factor 4F complex (eIF4F) assembly^21,23^. The latter complex is comprised of eIF4E (the cap-binding protein), eIF4G (a scaffold protein) and eIF4A (a DEAD-box helicase). Two associated proteins, eIF4B and the functionally related eIF4H, anchor the complex to the mRNA and increase eIF4A helicase activity. Dysregulation of eIF4F complex members drives tumorigenesis via increased synthesis of proteins that are normally poorly translated, particularly those encoding growth factors and oncogenes^24–26^. In addition, aberrant signalling via the PI3K-mammalian/mechanistic target of rapamycin (mTOR) axis activates protein synthesis, through increased phosphorylation of the components that regulate eIF4F complex assembly (e.g., 4EBPs and PDCD4) and ribosomes^24,27^ and interestingly, a number of studies have identified this pathway as being upregulated in MpM^20,28–30^. There are data to suggest that the product of *NF2*, Merlin, inhibits mTORC1 signalling, providing a rationale as to why this pathway is upregulated in *NF2*-deficient MpM, although the molecular mechanisms that link these two events have not been elucidated^31^.

Here, we show that in MpM there is a selective increase in the translation of mRNAs encoding proteins required for ribosome assembly and mitochondrial biogenesis, and this results in an enhanced global rate of mRNA translation, abnormal mitochondrial morphology and oxygen consumption, and reprogramming of metabolic outputs. However, inhibition of mRNA translation by pharmacological targeting of mTORC1 and 2, reverses these changes both in vitro and in ex vivo patient tumour tissue. Moreover, such mTORC1 and 2 inhibition extends survival in animal models of asbestos-induced mesothelioma and taken together, our data suggest that targeting mRNA translation could provide a viable, alternative treatment route for this incurable disease.

## Results

### Aberrant mRNA translation leads to upregulation in the synthesis of mitochondrial proteins and components of the translation apparatus

In previous studies, we established a series of patient-derived primary mesothelioma cell lines that have characteristics that are representative of MpM tumours with loss of *NF2* (merlin) and *CDKN2A*^32^. Importantly, these primary cell lines also show a degree of molecular diversity, thus capturing the disease heterogeneity in a patient cohort^32^. MpM has no known oncogenic drivers, therefore, we used these cells to determine changes to the translatome; cell lines used included Meso 3 T, 7 T, 8 T, 9 T, 12 T, 14 T and 17 T as described^32^, in addition to 13 T which was generated at the same time, and has the same MpM characteristics. Peritoneal mesothelial cells either from a single healthy individual (NM) or a pool of healthy donors (NMS) were used as controls.

**Fig. 1:**
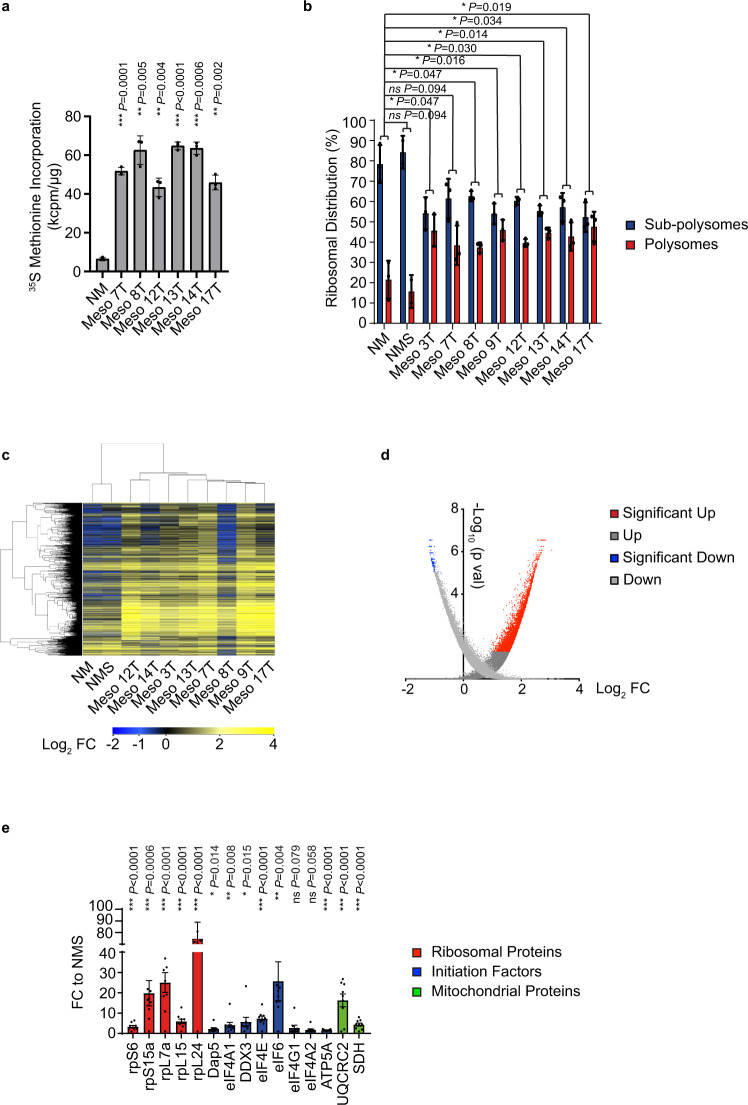
Aberrant translational regulation increases the synthesis of ribosomal proteins, protein synthesis machinery and mitochondrial components. **a** To assess protein synthesis rates, healthy mesothelial cells (NM) and primary MpM-derived cell lines were incubated with 35 S methionine and the rate of incorporation over a 30 min period was assessed. Error bars represent standard deviation and the measure of the centre of the error bars is the mean (*n* = 3, where *n* = number of biological repeats of 35 S methionine incorporation experiment) and significance was assessed using two-sided unpaired student’s *t* test with multiple comparisons, *p* values are shown on the bar graphs. **b** Polysome profiles of either healthy mesothelial cells (NM and NMS) or MpM-derived cell lines were obtained (*n* = 3, representative profile shown in Supplementary Fig. 1B, where *n* = number of biological repeats of the polysome profile experiment) and the area attributed to the subpolysomes (blue; subpolysomes fraction contains the 40 S, 60 S SUs and 80 S ribosomes and are generally not associated with translating mRNAs) or polysomes (red, polysomes are associated with translating mRNAs) calculated. Error bars represent standard deviation and the measure of the centre of the error bars is the mean (*n* = 3). Significance was assessed using a two-sided unpaired student’s *t* test with multiple comparisons, *p* values are shown on the bar graphs. **c** Polysome profiling was carried out using eight primary tumour cell lines and two controls with three biological repeats of each tumour cell or control cells used. Translation efficiency pattern and hierarchical clustering of control (NM–cells from a single donor and NMS–pool from four donors) and eight primary mesothelioma cell lines (*n* = 3 in each case). The heatmap displays the polysomal association of the most variable transcripts across the samples (fold change >2 compared with NM control). The legend bar shows the colour code for the normalised log intensity values. The hierarchical clustering shows the measure of the Pearson Correlation distance. **d** Volcano plot representing in red the mRNAs with the significant polysomal association and in blue a minority of downregulated transcripts. Polysome profiling array was analysed by RankProd, which generates two lists: the UP list (dark grey) contains every gene, their FC and their probability of being truly upregulated, and the DOWN list (light grey), with every gene, their FC and their probability of being truly downregulated. Each gene was included twice. Specifically, one arm of the plot includes all the genes with their probability of being UP, and the other arm of the plot includes all the genes and their probability of being DOWN regulated. **e** Densitometric analysis of the western blots in Supplementary Figs. 2C–E. Protein levels in MpM-derived cell lines are compared with NMS control cells. Error bars represent standard deviation and the measure of the centre of the error bars is the mean (*n* = 8 independent biological repeats) and significance was assessed using unpaired two-sided student’s *t* test adjusted for multiple comparisons, *p* values are shown on the bar graphs. Ribosomal factors are shown in the red bars, initiation factors in blue and mitochondrial proteins in green.

The majority of tumours display increased protein synthesis rates^22^ and consistent with these findings, both methionine incorporation assays (Fig. 1a) and puromycin labelling (Supplementary Fig. 1A) showed that MpM-derived tumour cells have greatly elevated global levels of mRNA translation. Moreover, sucrose density gradient analysis (Supplementary Fig. 1B), which allows the separation of actively translating ribosomes (polysomes) from subpolysomal particles, shows an increase in the association of mRNAs with the polysome in MpM cells (Fig. 1b, Supplementary Fig. 1B). In addition to a global upregulation of protein synthesis, the data suggest that most cancers additionally have increased translation of specific mRNAs^22^. To identify the mRNAs that were selectively translated in MpM, polysome profiling was performed. This technique allows the identification of candidate mRNAs whose translational behaviour differs significantly from the translation pattern observed in the control cells. This was carried out using cytoplasmic extracts prepared from 8 MpM-derived primary cell lines (Meso 3 T, 7 T, 8 T, 9 T, 12 T, 13 T, 14 T and 17 T) and two controls of primary mesothelial cells obtained from a healthy individual (NM) or a pool of healthy donors (NMS). The data showed that the translatome of MpM-derived primary cell lines clustered together, distinct from the controls (Fig. 1c, Supplementary Fig. 1C) with a significant number of mRNAs showing alterations in the degree of polysomal association (Fig. 1d, Supplementary data 1; the number of genes significantly upregulated was 1665, whereas 282 were downregulated) as confirmed by qPCR (Supplementary Fig. 2A, B). The transcripts that were significantly upregulated were classified based on their biological function and the top upregulated GO terms included mitochondria, electron transport chain components, ribosomal proteins and canonical translational machinery (Table 1, Supplementary data 2). Importantly, these GO terms are distinct from those obtained when transcriptional profiling was carried out on these lines^32^. The mRNAs that were more polysomally associated had shorter and unstructured 5′-UTRs (Table 2) compared with the downregulated. Although some of the mRNAs found to be translationally upregulated contained a terminal oligopyrimidine track (TOP) motif, there was not a significant enrichment in this category of mRNAs (Table 2). We did not find any enrichment of internal ribosome entry sites^33^, however, for the mRNAs encoding mitochondrial proteins, there was an enrichment of the TISU sequence in the 5′-UTR^34^.

**Table 1:** Table showing the top 10 enriched clusters of highly translated mRNAs in MpM-derived cell lines.

No.	GO term	Description	Odds ratio	*P* value
1	GO:0005739	Mitochondrion	2.21	2.60E-13
2	GO:0022904	Respiratory electron transport chain	5.73	4.68E-10
3	GO:0016070	RNA metabolic process	3.52	2.93E-08
4	GO:0003735	Structural constituent of ribosome	4.27	4.68E-07
5	GO:0006412	Translation	3.45	1.05E-06
6	GO:0010467	Gene expression	2.36	3.56E-06
7	GO:0005743	Mitochondrial inner membrane	2.94	5.99E-03
8	GO:0016071	mRNA metabolic process	3.27	5.76E-02
9	GO:0016032	Viral reproduction	2.77	9.52E-02
10	GO:0000216	M/G1 transition of mitotic cell cycle	4.87	1.77E-01

Gene Ontology terms (Biological Processes) were used for the analysis. The most enriched clusters were related to mitochondria and protein synthesis.

**Table 2:** Structural features of the mRNAs that are most upregulated.

Description			*P* value
shorter 5′-UTR	*t* test	***	2.805E-08
higher 5′-UTR MFE	*t* test	***	1.558E-11
fewer uORF	*χ*^2^	***	1.096E-07
TISU elements	*χ*^2^	***	6.074E-06
TOP/TOP like	*χ*^2^	ns	
AU-rich elements	*χ*^2^	ns	

These included upstream open-reading frame (uORF) = 833; terminal oligopyrimidine tract TOP/TOP-like = 126; AU-rich elements =  297; translation initiator of short 5′-UTR, TISU = 28 MFE = minimum-free energy.

Western blot analysis to confirm changes in expression of proteins that form part of the ribosomes (Fig. 1e and Supplementary Fig. 2C) identified large increases in the expression of selected ribosomal proteins in both the small and large subunit proteins, ranging from a threefold change in rpS6 to over 80-fold for rpL24 (Fig. 1e). We also identified increased expression of canonical and non-canonical RNA binding proteins that control translation, e.g., increased expression of eIF4A1, DAP5 (which is encoded by eIF4G2) and DDX3 (Fig. 1e and Supplementary Fig. 2D). We additionally identified increases in eIF6 and eIF4E, in agreement with previous studies^35,36^ (Fig. 1e and Supplementary Fig. 2D). Proteins required for mitochondrial oxidative phosphorylation, e.g., mitochondrial ATP synthase (ATP5A) and ubiquinol-cytochrome c reductase complex (UQCR2) and the TCA cycle, including succinate dehydrogenase (SDH) (Fig. 1e and Supplementary Fig. 2E), were also confirmed as upregulated by western blot analysis. As controls, we used eIF4G1 or eIF4A2 that showed no change agreement with the array data and neogenin a protein involved in neurogenesis (Supplementary Fig. 2B, F); which was downregulated, in agreement with the profiling data.

**Fig. 2:**
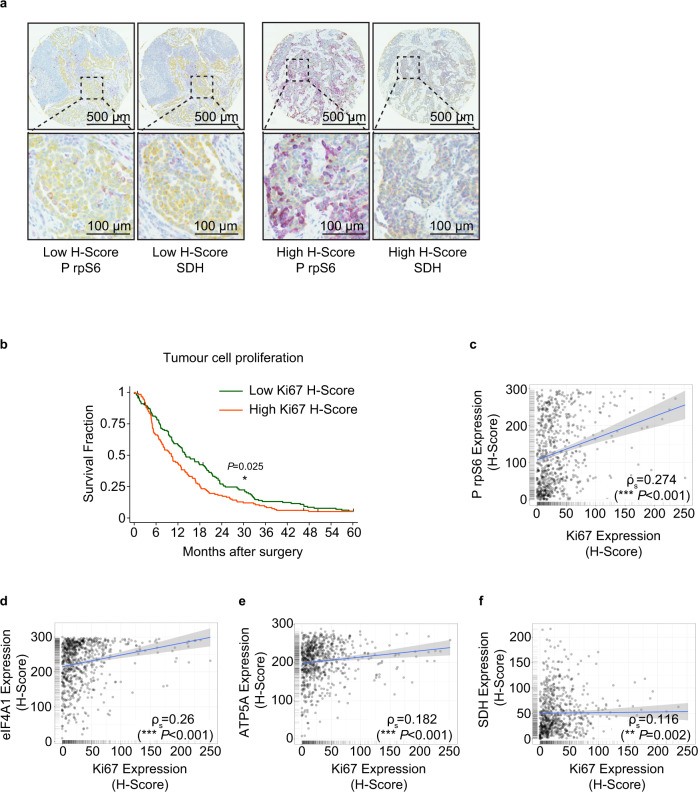
Protein synthesis and mitochondrial protein expression correlate with poor survival in patient-derived material. **a** Representative section of tissue microarray (TMA) probed with antibodies against phospho-Ser240/244 rpS6 (magenta) and SDH (magenta). Tumour areas within the TMA cores were identified by cytokeratin staining (yellow). Visiopharm® software was used to quantify the magenta staining in the tumour area. **b** Kaplan–Meier plot, showing Ki67 staining is a strong predictor of survival. The 272 patients included in the TMAs, were split into two equally sized groups according to Ki67 H-score. A log-rank test was performed to determine the significance of differences in survival (*p = 0.025). **c** Scatter plot of the H-scores of rpS6 phosphorylation (mTOR activity) and Ki67 expression in mesothelioma TMAs. Linear regression analysis was performed by Spearman’s rank correlation, the coefficient was 0.274. The linear regression fit is depicted with a blue line. The grey band shown is the 95% confidence interval. **d** Scatter plot of the H-scores of eIF4A1 expression and Ki67 expression in mesothelioma TMAs. Linear regression analysis was performed by Spearman’s rank correlation, the coefficient was 0.26. The linear regression fit is depicted with a blue line. The grey band shown is the 95% confidence interval. **e** Scatter plot of the H-scores of mitochondrial ATP5A expression and Ki67 expression in mesothelioma TMAs. Linear regression analysis was performed by Spearman’s rank correlation, the coefficient was 0.182. The linear regression fit is depicted with a blue line. The grey band shown is the 95% confidence interval. **f** Scatter plot of the H-scores of mitochondrial SDH expression and Ki67 expression in mesothelioma TMAs. Linear regression analysis was performed by Spearman’s rank correlation, the coefficient was 0.116. The linear regression fit is depicted with a blue line. The grey band shown is the 95% confidence interval.

We then asked whether there was a correlation between tumour cell growth and the activation of mRNA translation and expression of mitochondrial proteins. This was achieved by generating a tissue microarray (TMA), comprised of 272 archival formalin-fixed surgical tumour samples from individuals with mesothelioma (Supplementary Fig. 3A). These data showed, in agreement with previous studies, that epithelioid histology has a better outcome than sarcomatoid tumours (Supplementary Fig. 3B). The TMAs were probed with antibodies against Ki67 to assess tumour cell proliferation and selected proteins identified as upregulated in the primary MpM-derived cell lines, including eIF4A, rpS6, ATP5A and SDH (Fig. 1e and Fig. 2a). Quantitative protein expression data were obtained from images using Visiopharm® software and a tumour cell cytoplasm-specific H-score was obtained using duplex staining for cytokeratin expression. The automated method was validated against manual scoring (Supplementary Fig. 3C, D). As expected, the data show that patients with a high expression of the proliferation marker Ki67 have a significantly worse overall survival rate (Fig. 2b). Importantly, there were significant positive associations between expression of Ki67 and P rpS6, SDH, eIF4A and ATP5A, consistent with a link between or co-regulation of the processes of protein synthesis, mitochondrial function and enhanced cell growth in MpM tumours (Fig. 2c–f).

### Mitochondria in MpM show distinct morphological changes

**Fig. 3:**
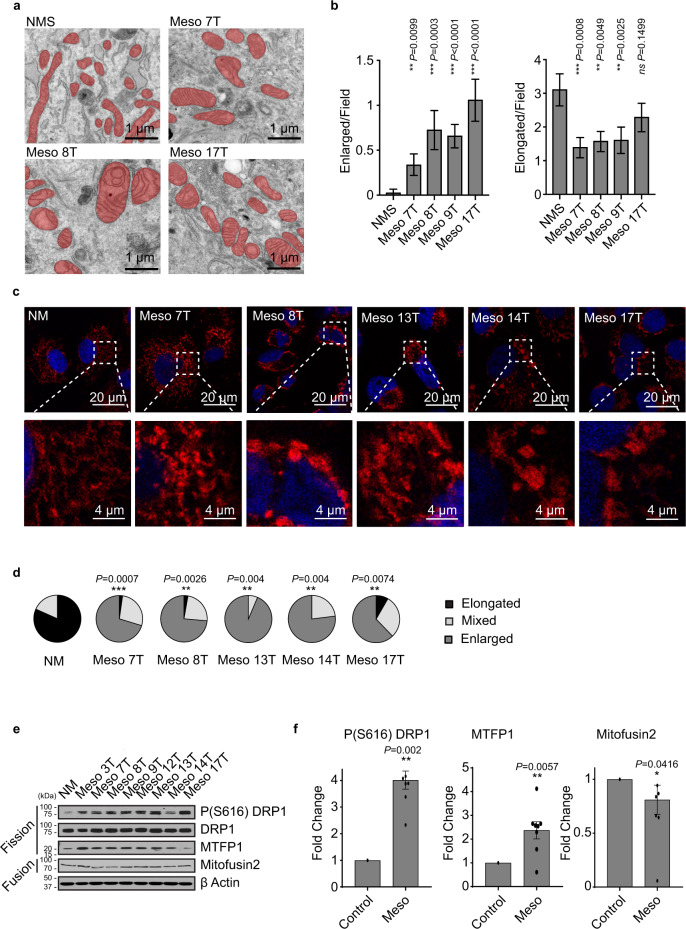
Aberrant synthesis of mitochondrial components and alterations in phosphorylation of fission proteins changes mitochondrial morphology and function. **a** Representative electron micrographs of primary MpM cell lines (Meso 7 T, Meso 8 T, Meso17T) and control cells (NMS). Scale bar 1 μm. **b** The average number of enlarged particles per field. In all, 19–23 different micrographs (for *n* = 369 mitochondrial particles) were analysed per sample. Error bars represent SD and significance was assessed using Mann–Whitney test, *p* values are shown on the bar graphs. The average number of elongated particles per field. In all, 19–23 different micrographs (*n* = 369 mitochondrial particles) were analysed per sample. Error bars represent SEM and the measure of the centre of the error bars is the mean, significance was assessed using Mann–Whitney test *p* values are shown above the bars. **c** Confocal images of primary MpM cells or healthy mesothelial control, stained with DAPI (blue-nuclei) and ATPB (red-mitochondria). Scale bar 20 μm or 4 μM for the call outs. **d** The mitochondrial networks in the samples from **c** were assessed in terms of the extent of elongated, mixed or enlarged/fragmented mitochondria and the data represented as pie charts, (black sections = elongated, light grey sections = mixed and dark grey = enlarged). The significance of changes in the elongated mitochondrial network was assessed using two-sided unpaired Student’s *t* test NM vs tumour cell lines. *P* values are shown above the pie charts. **e** Western blot analysis of eight primary cell lines derived from patients with MpM and from untransformed mesothelium (NM), which were probed with antibodies against phospho-Ser616 DRP, DRP1, MTPF1 and Mitofusin2. Beta-actin was used as a sample integrity control. Source data are provided as a Source Data file. **f** Densitometric analysis of the western blots in data shown in Fig. 2e. Protein levels in MpM-derived cell lines are compared to NM control cells. Error bars represent standard deviation, and the measure of the centre of the error bars is the mean (*n* = 8 biological repeats). Significance was assessed using a two-sided unpaired student’s *t* test with multiple comparisons, *p* values are shown on the bar graphs.

Given the unexpected selective upregulated synthesis of nuclear-encoded proteins with mitochondrial functions, we also assessed whether there were changes in mitochondrial morphology, function and metabolism. Electron microscopy revealed significant ultrastructural alterations to mitochondria in MpM-derived cell lines (Fig. 3a), with rounded and enlarged 2D cross-sectional appearances (Fig. 3b and Supplementary Fig. 4). Mitochondria form a dynamic network, changes in which are associated with a range of diseases^37^. To assess alterations in the mitochondrial network, confocal microscopy was used (Fig. 3c). In control cells, the mitochondria form an elongated network, whereas in MpM cells mitochondria were fragmented (Figs. 3c, d). Mitochondrial dynamics are controlled by mitochondrial fission process 1 (MTFP1), which couples pro-fission phosphorylation (Ser616) and mitochondrial recruitment of dynamin-related protein 1 (DRP1), whereas fusion is regulated by mitofusion 1 and 2. Significantly increased expression of the pro-fission protein MTFP1 and enhanced phosphorylation of DRP1 were detected in MpM cells (Fig. 3e, f), which were consistent with the changes in mitochondrial shape observed in confocal and electron microscopy.

### Metabolic changes in aberrant MpM-derived mitochondria provide energy and metabolites for tumour cell growth

**Fig. 4:**
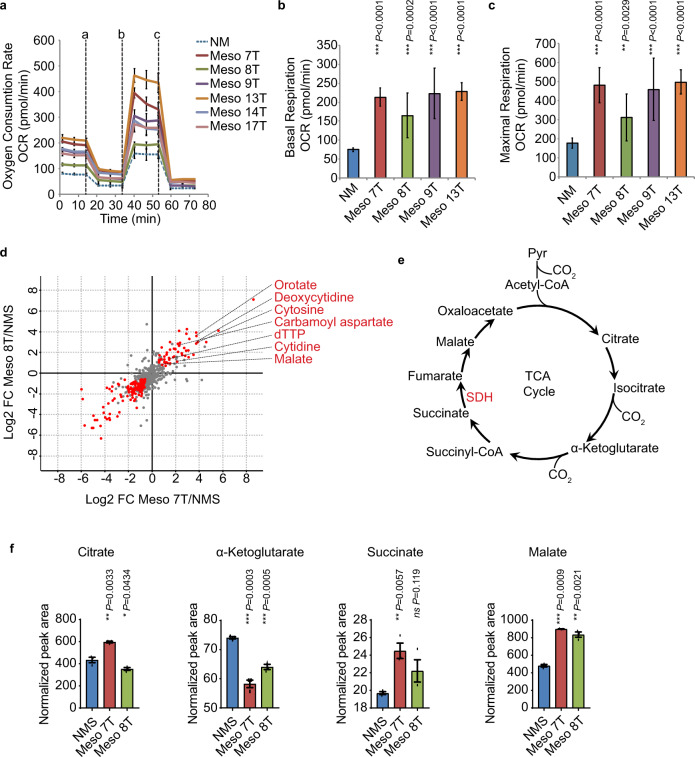
Metabolic changes in MpM-derived cells. **a** The oxygen consumption rate (OCR) of primary MpM and NM control cells was measured in the seahorse extracellular flux assay. The vertical dashed lines indicate when oligomycin (**a**), FCCP (**b**) and rotenone + antimycin A (**c**) were injected. Error bars represent standard deviation and the measure of the centre of the error bars is the mean (*n* = 6 where *n* = number of independent biological repeats of the oxygen consumption rate experiments). **b** The basal respiration of primary MpM and NM control cells was measured as the OCR value before oligomycin injection. Error bars represent standard deviation and the measure of the centre of the error bars is the mean. Significance was assessed using a two-tailed unpaired Student’s *t* test adjusted for multiple comparisons (*n* = 6 where *n* = number of independent biological repeats of the basal respiration rate measurements, *p* values are shown on the bar graphs). **c** The maximal respiration of primary MpM and NM control cells was measured as the highest OCR value after FCCP injection. Error bars represent standard deviation and the centre of the error bars is the mean. Significance was assessed using unpaired two-sided student’s *t* test adjusted for multiple comparisons (*n* = 6, *n* = number of biological repeats of maximal respiration rate measurements, *p* values are shown on the bar graphs). **d** Cell extracts were prepared from Meso 7 T, Meso 8 T and NMS cells and analysed by LC-MS/MS. Untargeted data analysis revealed increased intracellular abundance of malate, various intermediates of de novo pyrimidine synthesis, and derivatives of pyrimidine nucleotides in mesothelioma cells. Red dots indicate metabolites for which intracellular abundance was altered significantly and coherently in both cell lines compared with NMS cells (absolute log2 fold change ≥0.58; adjusted *p* value < 0.05 (Benjamini–Hochberg), ANOVA). **e** Schematic to show the TCA cycle. **f** Data analysis was performed to determine the intracellular abundance of citrate, α-ketoglutarate, succinate and malate. Data represent the mean ± SD obtained and the centre of the error bars represents the mean. Significance was assessed using two-sided unpaired two-sided student’s *t* test adjusted for multiple comparisons, *p* values are shown on the bar graphs. *NMS* = normal mesothelial cells, blue bars, Meso 7 T is red bars and Meso 8 T are the green bars.

To assess whether the differences in mitochondrial shape and network in MpM are associated with alterations in oxidative phosphorylation, oxygen consumption rate (OCR) was measured (Fig. 4a). The data show that basal (Fig. 4b) and maximum OCR (Fig. 4c) were raised in MpM-derived cell lines relative to the control, suggesting that upregulation of oxidative phosphorylation provides energy to drive the synthesis of proteins and organelles required for the high rate of tumour cell proliferation.

We then asked whether there were changes in the steady-state levels of metabolites involved in major bioenergetic pathways required to drive cell growth in MpM. Cells extracts were prepared from two mesothelioma-derived cell lines (Meso 7 T and 8 T) and NMS controls and subjected to metabolomics using liquid chromatography with tandem mass spectrometry (LC-MS/MS) (Fig. 4d). Guided by the data from an initial unbiased analysis which indicated changes in malate and nucleotides (Fig. 4d), we further interrogated TCA cycle intermediates and nucleotide biosynthesis. Changes in the TCA cycle (Fig. 4e) were of particular interest owing to the specific translational upregulation of SDH in MpM-derived cells (Fig. 1h). Guided by the data from the untargeted analysis, a targeted approach was applied to further interrogate pathways of interest (i.e. TCA cycle and nucleotide biosynthesis). The data show while there are modest differences in the abundance of the metabolites citrate, alpha ketoglutarate and succinate, there is a twofold increase in the intracellular abundance of the metabolite malate (Fig. 4f), which is downstream of SDH that we show to be translationally upregulated. There was also an increase in intermediates required for the production of purines and pyrimidines, such as carbamoyl aspartate and dihydroorotate (Supplementary Fig. 5, Supplementary data 3). Stable isotope labelling using ^13^C6-glucose and ^13^C5-glutamine revealed glutamine to be the major carbon source for TCA cycle intermediates and aspartate, with increased labelling being observed in Meso 7 T and Meso 8 T compared with NMS cells (Supplementary Fig. 6A, B). Pronounced labelling of carbamoyl aspartate was observed from ^13^C5-glutamine, but not from ^13^C4-aspartate, highlighting the relevance of de novo aspartate synthesis for nucleotides in these cells (Supplementary Fig. 6C).

### Targeting mRNA translation reverses the phenotype of MpM

**Fig. 5:**
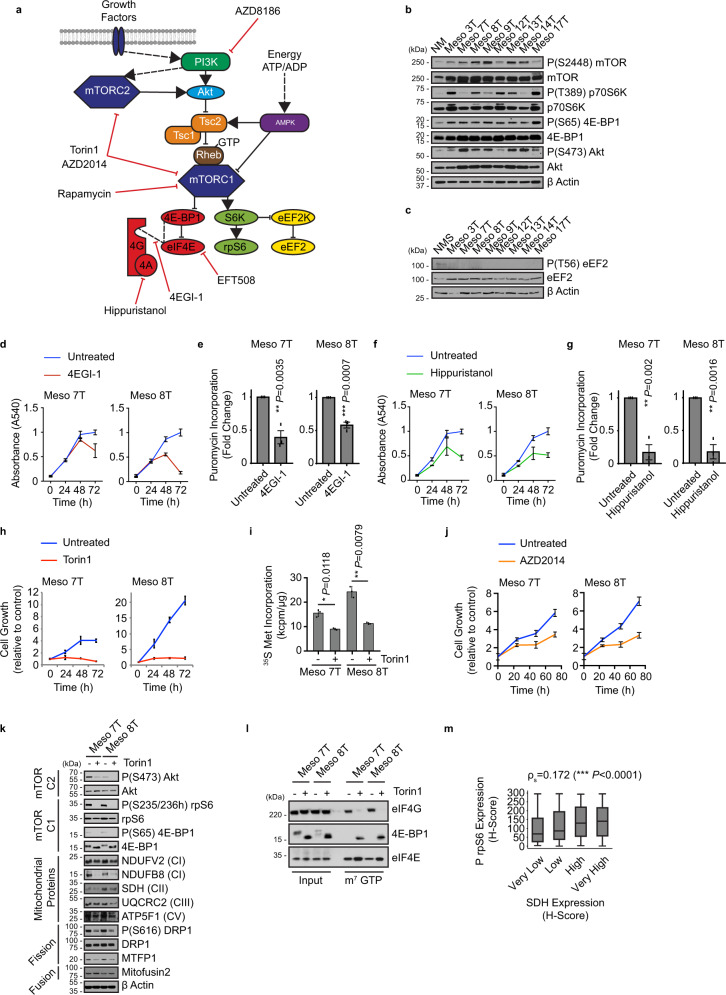
Aberrant signalling through mTOR correlates with increased protein synthesis in MpM. **a** Schematic summary showing mTORC1 and mTORC2 signalling pathways upstream of protein synthesis regulation. **b** Western blot analysis of eight primary cell lines derived from patients with MpM and from untransformed mesothelium (NM), which were probed with antibodies against Akt and phosphorylated Akt, as a marker for mTORC2 activity, and 4EBP1 and phosphorylated 4EBP1, p70S6K and phosphorylated p70S6K, as a marker for mTORC1 activity. Actin was used as a sample integrity control. Source data are provided as a Source Data file. Experiments were repeated on three occasions and similar data were obtained in each case. **c** Western blot analysis of eight cell lines derived from patients with MpM and from a pool of untransformed mesothelium (NMS), which were probed with antibodies against eEF2 and phosphorylated eEF2. Beta-actin was used as a sample integrity control. Experiments were repeated on three occasions. **d** Primary MpM cells (Meso 7 T or Meso 8 T) were treated with 25 μM 4EGI-1 and the rate of cell growth was assessed over a 72 h time period with a crystal violet assay. Error bars represent standard deviation, the centre of the error bars is the mean (*n* = 4 where *n* = number of biological repeats of growth rate measurements as determined by change in absorbance). Blueline is untreated, red line treated with 25 μM 4EGI-1. **e** Primary MpM cells (Meso 7 T or Meso 8 T) were treated with 25 μM 4EGI-1. Puromycin incorporation from *n* = 3 independent experiments (representative in Supplementary Fig. 7 A). Error bars represent standard deviation and significance was assessed using unpaired Student’s *t* test, *p* values are shown on the bar graphs. **f** Primary MpM cells (Meso 7 T or Meso 8 T) were treated with 100 nM Hippuristanol and the rate of cell growth was assessed over a 72 h time period with a crystal violet assay. Error bars represent standard deviation and the centre of the error bars represents the mean (*n* = 4 where *n* = number of biological repeats of growth rate measurements). Blueline is untreated, red line treated with 100 nM Hippuristanol. **g** Primary MpM cells (Meso 7 T or Meso 8 T) were treated with 100 nM Hippuristanol. Puromycin incorporation from *n* = 3 independent experiments (representative in Supplementary Fig. 7 C). Error bars represent standard deviation and the centre of the error bars represents the mean. Significance was assessed using two-sided unpaired Student’s *t* test adjusted for multiple comparisons, *p* values are shown on the bar graphs. **h** Primary MpM cells (Meso 7 T or Meso 8 T) were treated with 100 nM Torin 1 and the rate of cell growth was assessed over a 72 h time period with a crystal violet assay. Error bars represent standard deviation, with the centre of the error bar the mean (*n* = 3, *n* = number of independent biological repeats of growth rate measurements). Blueline is untreated, red line treated with 100 nM Torin 1. **i** To assess protein synthesis rates, primary MpM-derived cell lines Meso 7 T and Meso 8 T were incubated with 100 nM Torin 1 for 30 m, followed by 33 μCi of 35 S methionine and the rate of incorporation over a 30 m period was assessed. Error bars represent standard deviation and the centre of the error bars represents the mean. (*n* = 3) and where *n* = number of independent biological repeats. Significance was assessed using a two-sided unpaired Student’s *t* test adjusted for multiple comparisons, *p* values are shown on the bar graphs. **j** Primary MpM cells (Meso 7 T or Meso 8 T) were treated with 100 nM AZD2014 and the rate of cell growth was assessed over a 72 h time period with a crystal violet assay. Error bars represent standard deviation, the centre of the error bars represents the mean (*n* = 8, *n* = number of independent biological repeats of growth rate measurements). Blueline is untreated, red line treated with 100 nM AZD2014. **k** Western blot analysis of primary MpM cell lines (Meso 7 T and Meso 8 T) treated with 100 nM Torin 1 for 24 h and probed with antibodies to assess changes in mTOR pathway, mitochondrial protein expression and mitochondrial dynamics. Actin was used as a sample integrity control. **l** m7GTP pull-down assay was performed on primary MpM cell lines (Meso 7 T and Meso 8 T), which were treated with 100 nM Torin 1 for 30 m. Input extracts and cap analogue pull-downs were probed with antibodies against eIF4G and 4EBP1. eIF4E was used as an input and pull-down control. Experiments were repeated on three occasions. **m** Correlation analysis between rpS6 phosphorylation (mTOR activity) and SDH expression in mesothelioma TMAs. Of the *n* = 812 individual TMA cores, 723 (89.04%) had a Phosho rpS6 H-score and 734 (90.39%) had an SDH H-score. SDH H-score was split into four equally sized groups and Spearman’s correlation analysis was performed for Phospho pS6 H-score against these groups. From *n* = 689 cores which had both a pS6 and SDH H-score, Spearman’s correlation coefficient was 0.172. The box plots show the box from the first quartile to the third quartile. The middle line represents the median. The whiskers go up to the maximum and minimum values.

It was important to establish whether the upregulation of mitochondrial protein expression and function was the direct result of increased expression/activity of the canonical translation machinery. Such information would provide a way to target MpM tumour cell growth by limiting the enhanced metabolism and the global increase in protein synthesis rates, in addition to the selective targeting of specific nuclear-encoded mitochondrial proteins. To identify potentially targetable dependencies in the translation cascade we used a pharmacological approach to inhibit upstream pathways that signal to translation, in particular the mTOR axis^38^ or the direct inhibition of activity/function of eukaryotic initiation factors (Fig. 5a), that we identified as upregulated in MpM (Figs. 5b, c, Fig. 1e, Supplementary Fig. 2D). To inhibit the canonical translation machinery in MpM, cells were treated with hippuristanol to target eIF4A^39^, 4EGI-1^40^ to target the interaction of eIF4E with eIF4G, and the MNK inhibitor EFT508 (Tomivosertib)^41^ to inhibit eIF4E phosphorylation (Fig. 5a). Upstream signalling to translation was inhibited using rapamycin to block mTORC1 or mTORC1 and 2 combined (using Torin 1 or the clinically relevant AZD2014^42,43^) (Fig. 5a). The data show that directly targeting the canonical translation machinery using either 4EG-1 or hippuristanol decreased protein synthesis rates and reduced cell growth (Fig. 5d–g, Supplementary Fig. 7A, C). Treatment with EFT508 to inhibit MNK activity had no effect on protein synthesis or cell growth (Supplementary Fig. 7E–G). Inhibition of mTORC1 alone with Rapamycin (Supplementary Fig. 8A) had no significant effect on global protein synthesis rates in MpM cells (Supplementary Fig. 8B) recapitulating the ineffectiveness of the rapalog everolimus in phase II clinical trial^44^. However, dual targeting of mTORC1 and 2 with Torin1 resulted in a significant reduction in protein synthesis rates and cell growth (Fig. 5h, i, Supplementary Fig. 8C), with similar effects growth observed with AZD2014 (Fig. 5j, Supplementary Fig. 8D). These data are consistent with active-site mTOR inhibitors such as Torin1 or AZD2014, suppressing rapalog-resistant mTORC1 outputs, e.g., phosphorylation of 4E- BP1^45,46^.

We then assessed whether either direct or indirect targeting of mRNA translation would reduce the abnormal expression of mitochondrial proteins, which were selectively translationally upregulated. The data show that combined inhibition of TORC1 and 2 has a greater inhibitory effect on the synthesis of selected proteins and in addition, decreased the phosphorylation status of the pro-fission protein DRP1 and expression of MTFP1 (Fig. 5k, Supplementary Fig. 8E, F, Supplementary Fig. 9). There was a decrease in SDH expression following treatment of cells with 4EGI-1 (Supplementary Fig. 7B), but no change following treatment with hippuristanol or EFT508 (Supplementary Fig. 7D, H). Taken together, these data suggest that translation of selected mitochondrial proteins is particularly dependent on the eIF4E and eIF4G interaction, which is inhibited by targeting mTORC1 and 2 (Fig. 5l, Supplementary Fig. 9) or using 4EGI-1. Although there was a strong effect on cell growth with mTORC1 and 2 inhibition, this did not induce apoptosis (Supplementary Fig. 10). To address whether mTOR signalling correlated with the expression of mitochondrial proteins in human tumour material, tumour TMAs were probed with antibodies directed against phosphorylated rpS6 and SDH and the data show a highly significant positive correlation (Fig. 5m).

### Inhibition of translation reverses mitochondrial morphology and changes metabolic outputs

**Fig. 6:**
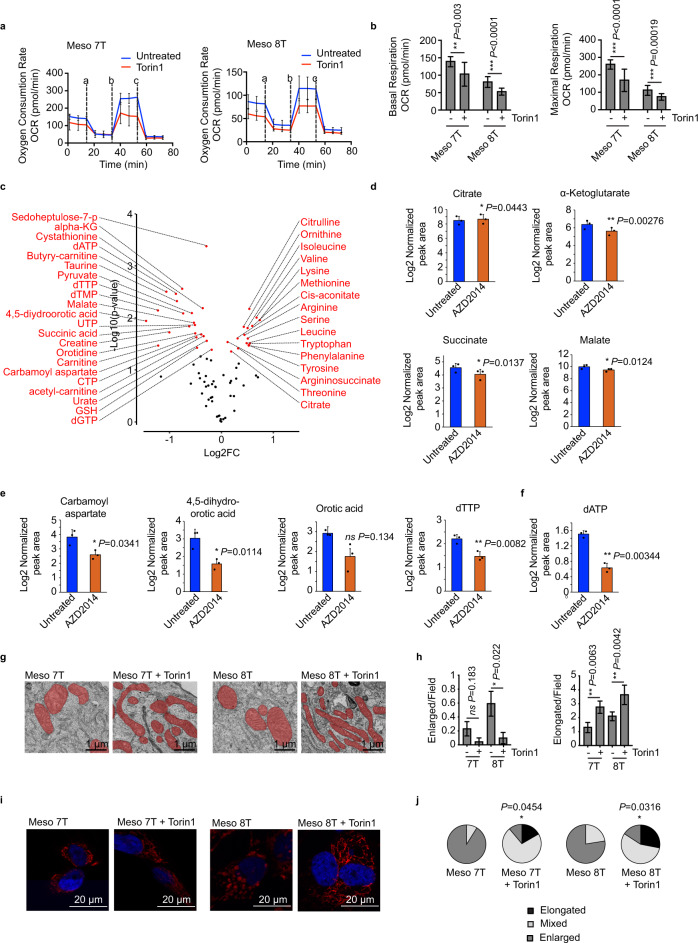
TORC1 and 2 inhibition reverse metabolic effects. **a** Primary MpM cells Meso 7 T and Meso 8 T were treated with 100 nM Torin1 or left untreated for 18–20 h. The oxygen consumption rate (OCR) was measured in the SeaHorse extracellular flux assay. The vertical dashed lines indicate when oligomycin (**a**), FCCP (**b**) and rotenone + antimycin A (**c**) were injected. Error bars represent standard deviation, the centre of the error bar is the mean (*n* = 6 independent biological repeats). **b** The basal respiration from A was measured as the OCR value before oligomycin injection. The maximal respiration from A was measured as the highest OCR value after FCCP injection. Error bars represent standard deviation and the centre of the error bar is the mean. Significance was assessed using two-sided unpaired student’s *t* test adjusted for multiple comparisons, *n* = 6 independent biological repeats. *P* values are shown on the bar graph. **c** Cell extracts were prepared from Meso 8 T cells treated with vehicle or 100 nM AZD2014 for 24 h to inhibit mTORC1 and mTORC2, and analysed for metabolites representing multiple metabolic pathways. Volcano plot showing changes in intracellular metabolite abundance upon treatment with AZD2014. Red dots indicate metabolites for which intracellular abundance was altered significantly. Three biological experiments, each with three technical replicates absolute log2 fold change ≥0.58; two-sided paired *t* test was used with Benjamini–Hochberg correction to log2 transformed and centred data using R. **d** Intracellular abundance of TCA cycle metabolites: citrate, α-ketoglutarate, succinate, malate. Data represent the mean ± SD obtained from three biological experiments, each with three technical replicates. Significance was assessed using a two-sided Student’s *t* test with Benjamini–Hochberg correction, *p* values are shown on the graph. Blue bars untreated, red bars treated with 100 nM AZD2014. **e** Intracellular abundance of pyrimidine synthesis intermediates: carbamoyl aspartate, dihydroorotate, orotate, dTTP. Data represent the mean ± SD obtained from three biological experiments, each with three technical replicates. Significance was determined using two-sided Student’s *t* with Benjamini–Hochberg correction, *p* values are shown on the graph. Blue bars untreated, red bars treated with 100 nM AZD2014. **f** Intracellular abundance of dATP. Data represent the mean ± SD obtained from three biological experiments, each with three technical replicates. Significance was assessed using paired two-sided student’s *t* test with Benjamini–Hochberg correction, *p* values are shown on the graphs. **g** Electron micrographs of primary MpM cell lines (Meso 7 T and Meso 8 T) treated with 100 nM Torin 1 for 48 h. Scale bar 1 μm. **h** Enlarged and elongated mitochondria per field in G were calculated. In all, 19–23 different micrographs (for 369 mitochondrial particles) were analysed per sample. Error bars represent SEM and significance was assessed using a Mann–Whitney test, *p* values are shown. **i** Confocal images of primary MpM cells Meso 7 T and Meso 8 T treated with 100 nM Torin 1 for 24 h or left untreated, stained with DAPI (blue-nuclei) and ATPB (red-mitochondria). Scale bar 20 μm. Experiments were repeated on three independent occasions. **j** The mitochondrial networks in the samples from **i** were assessed in terms of the extent of elongated, mixed or enlarged/fragmented mitochondria and the data represented as pie charts, (black sections = elongated, light grey sections = mixed and dark grey = enlarged). The significance of changes in the elongated mitochondrial network was assessed using an unpaired two-sided student’s *t* test adjusted for multiple comparisons. Untreated vs Torin 1, *p* values are shown on the bar graphs.

To examine the metabolic effects of inhibition of mTORC1 and 2 on mitochondrial function, seahorse analysis was used to determine changes in oxidative phosphorylation. There was a decrease in OCR in cells, consistent with a reduction in mitochondrial function (Fig. 6A, B). We then explored whether this similarly changed the metabolic profile (Fig. 6c, Supplementary data 4). Metabolomics revealed that MpM-derived cell lines treated with mTORC1 and 2 inhibitors resulted in a reduction in malate and TCA-derived metabolites, consistent with the reduced mitochondrial respiration (Fig. 6d) and a decrease in nucleotides and their precursors (Fig. 6e, f). There were also significant increases in free intracellular amino acids, consistent with the inhibition of protein synthesis (Fig. 6c) and also likely to be influenced by induction of autophagy (although this has not been explored further).

To explore whether inhibition of translation reversed the other aberrant mitochondria characteristics observed in MpM, cells were treated with mTORC1 and 2 inhibitors. Thus, following treatment with Torin1 we observed a reduction in mitochondrial 2D section size (Fig. 6g, h; Supplementary Fig. 11), the restoration of the mitochondrial network and mitochondrial elongation (Fig. 6i, j).

### Inhibition of translation in ex vivo patient explants, and in vivo in mouse models, inhibits tumour cell proliferation

**Fig. 7:**
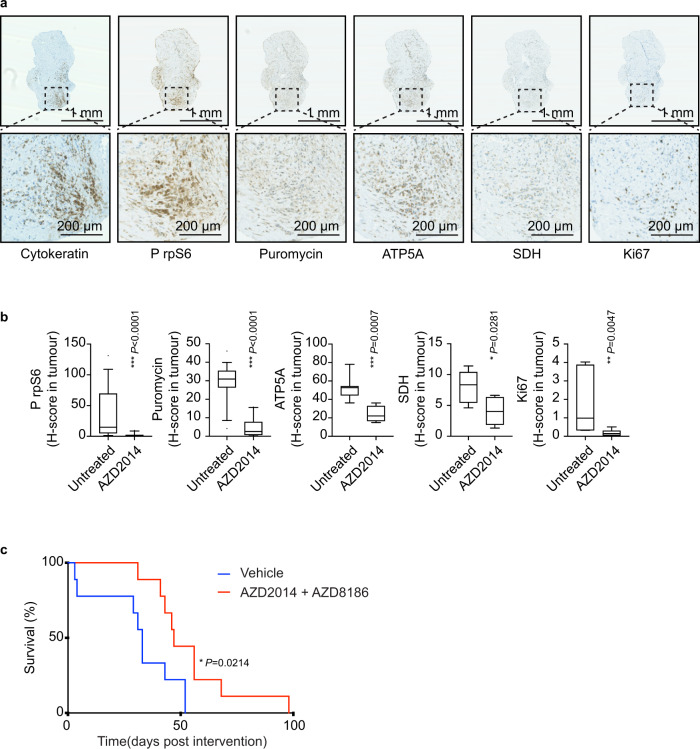
mTORC1 and 2 inactivation inhibits tumour cell growth ex vivo and in mouse models of MpM. **a** Representative sections of mesothelioma explants stained with the indicated antibody. Cytokeratin staining was used to identify tumour areas within the explants. Scale bar 1 μm, 200 μm for the call outs. **b** Mesothelioma explants were cultured for 72 h with 100 nM AZD2014 or with vehicle. H-score quantification of IHC staining within tumour areas is performed by Visiopharm® software. Mann–Witney significance test was performed (two patients, *n* = 8 explants, each *p* values are shown on the graph). The box plots show the box from the first quartile to the third quartile. The middle line represents the median. The whiskers go up to the maximum and minimum values. **c** Adult mice (*Cdkn2a*^*−/−*^*;Nf2*^*fl/fl*^*;Tp53*^*fl/fl*^) were intrapleurally injected with 107 pfu Lenti-Cre, followed after 10 days by a single intrapleural injection of 25 μg of long fibre amosite asbestos and allowed to age for a further 50 days. The mice were treated with AZD2014 + AZD8186 (*n* = 9 mice) or vehicle control (*n* = 9 mice), from d60 post-viral induction of floxed alleles. Graph shows survival from the first day of treatment (Mantel–Cox log-rank test).

We then explored the effect of translation inhibition on MpM tumour cell growth using ex vivo patient samples and in vivo mouse models of asbestos-induced MpM. To determine whether the inhibitory in vitro cell growth effects could be recapitulated ex vivo, mTORC1 and 2 were inhibited (using either AZD2014 or Torin 1) in explants from freshly resected human tumours; samples were stained for cytokeratin (as a marker of tumour area), puromycin (to assess protein synthesis) and phospho rpS6, ATP5A, SDH and Ki67 (Fig. 7a). Following inhibition of mTORC1 and 2 there was a decrease in protein synthesis rates in the tumour samples as measured using puromycin incorporation assays (Fig. 7b and Supplementary Fig. 12B), consistent with a change in the phosphorylation status of rpS6 (Fig. 7b and Supplementary Fig. 12A), and in parallel there was a decrease in expression of SDH and ATP5A (Fig. 7b and Supplementary Fig. 12C, D), and a concomitant decrease in tumour cell growth as shown by Ki67 staining (Fig. 7b and Supplementary Fig. 12E). Inhibition of eIF4A correlated with a reduction in staining for puromycin and Ki67, however, there was no decrease in ATP5A, SDH or phospho rpS6 (Supplementary Fig. 12F–J). Given these data, we reasoned that the most effective way to target mesothelioma in vivo was to simultaneously inhibit mTORC1 and mTORC2. In this regard, the dual mTORC1 and 2 inhibitor AZD2014 (vistusertib) has been trialled clinically^43,47,48^. However, as cancer cells can escape the requirement of signalling through mTOR by increasing nutrient uptake^49^ and inhibition of mTORC1 can result in feedback activation of the PI3K/AKT axis^50,51^ (Supplementary Fig. 12L), for longer-term targeting of this pathway in tumours it is advantageous to use this drug in combination with a PI3K inhibitor, such as AZD8186^42,52,53^ (Supplementary Fig. 12K), a combination that has been trialled clinically without unacceptable toxicity^54^. This combination is therefore most relevant in terms of putative human intervention strategies in the future. In agreement with previous studies, although AZD8186 has no effect on its own (Supplementary Fig. 12M, N) combining AZD2014 with AZD8186 increased the inhibition of signalling through mTORC1 and 2^52,53^, particularly at later time periods (Supplementary Fig. 12K–N; e.g., compare lane 12 and 13 in Supplementary Fig. 12L).

We then tested this combination for its potential therapeutic benefit in vivo, using a genetically engineered mouse model of Cre- inducible mesothelioma described previously^55^, which was adapted to include the disease-relevant asbestos-driven inflammatory landscape. Adult mice bearing homozygous floxed alleles for Tp53 and Nf2 and constitutively deleted for *Cdkn2a* (*Cdkn2a*^*−/−*^*; Nf2*^*fl/fl*^*; Tp53*^*fl/fl*^ termed “CNP”) were intrapleural injected with 107 pfu Lenti-Cre, followed after 10 days by a single intrapleural injection of 25 μg of long fibre amosite asbestos, and allowed to age for a further 50 days. The mice were treated with AZD8186 and AZD2014 combination^54^. Starting from day 60 post-Lenti-Cre induction, mice were treated daily with a combination of AZD8186 (50 mg/kg twice daily) and AZD2014 (15 mg/kg, once daily), or vehicle control, and maintained until symptoms required humane euthanasia. The combination of AZD2014 and AZD8186 yielded a significant extension of survival (Fig. 7c). Immunohistochemistry analysis of phospho rpS6 in the tumour region shows a reduction consistent with mTOR inhibition (Supplementary Fig. 13A, B).

Taken together, these data strongly suggest that inhibition of translation and energy production by targeting mTORC1 and 2 would restrict tumour cell growth in patients with MpM and provide a viable treatment option to extend lifespan, which is on average less than 18 months from diagnosis.

## Discussion

MpM is not associated with activation of any classical proto-oncogenic drivers, implicating epigenetic and regulatory mechanisms such as post-transcriptional dysregulation in the aberrant cell growth phenotype. Our data show that in MpM there is upregulation of global mRNA translation with a striking superimposed selective increase in the synthesis of ribosomal proteins, themselves central components of the canonical translational apparatus, and specific mitochondrial proteins (Figs. 1–2). This upregulation of mitochondrial and translational machinery proteins is seen in primary human tumour tissue, and both are related to tumour cell proliferation rates (Fig. 2). In MpM cells, changes in mitochondrial shape and connectivity were related to alterations in key Krebs cycle metabolites, which correlate with the synthesis of key metabolic enzymes (Figs. 3–4). In particular, increased expression of SDH correlates with enhanced production of TCA intermediates, leading to the production of pyrimidine and purines required for cell growth (Fig. 4). To identify ways in which to reduce mRNA translation and mitochondrial output, protein synthesis was targeted by either directly inhibiting the canonical translation machinery or upstream signalling pathways. Interestingly, drugs that reduce the binding of eIF4G to eIF4E, either through increased sequestration of eIF4E by eIF4E-BP1, or by direct interference decouple the link between translation, synthesis of ribosomal components and mitochondrial proteins (Figs. 4–6). Thus, while treatment with 4EGI-1, Torin1 and hippuristanol reduced global protein synthesis by ~50% and ~75%, respectively, the data show that treatment with either Torin1 or 4EGI-1 reduces the expression of SDH, (Fig. 5k, Supplementary Fig. 9, Supplementary Fig. 7B), whereas there was no effect with hippuristanol (Supplementary Fig. 7D), despite the greater reduction in global protein synthesis. Moreover, mTOR inhibition has a differential effect on the synthesis of additional mitochondrial proteins with a decrease in the expression of ATP5, and NDUFB8, but no effect in the expression of the fission protein DRP1 (Fig. 5k, Supplementary Fig. 9). Interestingly, the lack of reduction in expression of mitochondrial proteins in the presence of the eIF4A inhibitor hippuristanol is consistent with data that showed that selected mTOR-sensitive mRNAs encoding proteins with mitochondrial function had short 5′UTRs, and lacked a 5′ TOP motif and were relatively insensitive to eIFA4A1^56^. And additional studies, which showed that eIF4A depletion stimulates mTOR and does not affect the translation of mRNAs with short 5′ UTRs, including components of the electron transport chain that contain the TISU element^57^. It is likely that eIF4A reduces cell growth via additional mechanisms, and this is the subject of further studies.

These data are supported by earlier elegant studies in animal model systems that showed that mTORC1 controls mitochondrial activity and biogenesis by selectively promoting the translation of nuclear-encoded mitochondria-related mRNAs via inhibition of 4EBPs^58^. In addition, it has been shown that mitochondrial fission is linked by mTORC1 signalling to 4EBP1-mediated translational regulation of MTFP1^59^ and consistent with these studies, in MpM the mitochondria fission driven by mTORC1 and 2 correlates with DRP1 phosphorylation status (Figs. 3 and 5), and inhibition of this pathway significantly normalised the morphology of the mitochondrial network, and restored mitochondrial metabolism (Fig. 6). Crucially, we also demonstrate that combined mTORC1 and 2 inhibition impacts on cell growth of cultured and ex vivo tumour tissue and significantly extends the survival of mice with asbestos-induced mesothelioma (Fig. 7).

Development of MpM is associated with the loss of tumour suppressor genes, in particular, *NF2*^11,13–15^ and *CDKN2A*^11,17^ both of which were deleted/silenced in the primary cells used in our experiments, and there is evidence linking the loss of these to translational dysregulation in a way that is consistent with our observations. A number of studies have linked the loss of *NF2*, which encodes the protein Merlin, to increased signalling through mTOR^60–63^. Activation of signalling through mTOR would enhance protein synthesis rates, and given our data, the production of mitochondrial proteins to provide energy for this process. Epigenetic silencing and loss of *p19/Arf* precede the development of MpM in mice. Arf has a central role in the control of ribosome biogenesis and represses these processes either directly through its interaction with nucleophosmin^64,65^ or indirectly by controlling TTF-I localisation and repressing the transcription of rRNA genes^66^. A combination of loss of *Arf* and increased synthesis of ribosomal proteins identified in our study would increase net ribosome biogenesis, which is essential for tumour cell growth.

Taking the above into consideration, we suggest that the observed post-transcriptional cytoplasmic alterations in the MpM cells are the result of the loss of key tumour suppressor genes and facilitate tumour cell growth in the absence of classical proto-oncogenic drivers. Therefore, it is attractive to devise therapeutic regimes that target upregulated ribosome biogenesis, mRNA translation and the abnormal mitochondria which support these processes. Here, we show that combined inhibition of mRNA translation by mTORC1 and mTORC2 targeting significantly reduces tumour cell growth in cell lines and in ex vivo patient material and that a clinically relevant combination of the bispecific inhibitor AZD2014 with AZD8186 significantly extended survival of transgenic mice with MpM. Our data suggest that treatment with these drugs by targeting both mitochondrial fission/fusion and the selective synthesis of nuclear-encoded mitochondrial proteins and components of the canonical translation machinery reduces aberrant cell growth and leads to lifespan extension.

This biologically rational approach of the dual targeting of mTORC1 and mTORC2 offers hope for effective targeted therapy in this unique and devastating malignancy.

## Methods

All research was carried out in compliance with all relevant ethical regulations for animal testing and human research as detailed in the individual sections.

### Material availability

All unique/stable reagents generated in this study are available from the lead contact with a completed Materials Transfer Agreement.

### Cell culture

Mesothelioma-derived primary cells were isolated as described^32^ in addition to 13 T, which was derived at the same time using the same methodology and displays the same mesothelioma characteristics. Mesothelioma-derived primary cells were cultured in Roswell Park Memorial Institute Medium (RPMI)-1640 growth media supplemented with l-glutamine (2 mM), penicillin (100 U/ml), streptomycin (100 μg/ml), hEGF (20 ng/ml), hydrocortisone (1 μg/ml) and 10% FBS at 37 °C and 5% CO_2_. Primary untransformed mesothelium cells NM (single donor) and NMS (pool of four donors) were purchased from Zenbio and maintained according to the manufacturer’s indications. For all experiments, Mesothelioma primary tumour cells and untransformed mesothelium controls were plated in RPMI with l-glutamine (2 mM), 10% FBS, but with no additional growth factors and adapted at 37 °C and 5% CO_2_ for 18 h, before being treated as indicated. Cell growth was measured by crystal violet staining. Cells were plated in 96 wells at least *n* = 3 per condition. Cells were treated and then fixed in 3% paraformaldehyde (PFA) (Sigma), 2% sucrose in PBS 1× solution for 10 m at 25 °C, stained with 0.05% crystal violet, washed in water and dried at 25 °C. The dye was solubilized in 10% acetic acid and the absorbance at 540 nm was measured with a Power Wave XS2 plate reader (BioTek).

### 35 S methionine incorporation and puromycin incorporation

Cells were pulsed with ^35^S Met/Cys for 30 m at 37 °C in a 5% CO_2_ incubator. Met/Cys incorporation was stopped by chilled PBS wash. Cells were lysed in radioimmunoprecipitation assay buffer, and proteins were precipitated with Trichloroacetic Acid (TCA) ^35^S. Met/Cys incorporation into newly synthesised proteins was evaluated by scintillation counting. Bicinchoninic Acid (BCA) assay was performed on the sample extracts to normalise the protein concentration.

Puromycin incorporation into nascent polypeptides was performed according to the SUnSET method^67^. In brief, primary cells were incubated with 10 μg/ml of puromycin for 10 m, before being lysed and protein immunoblotted with 12D10 monoclonal antibody to puromycin (Millipore). Equal loading was visualised by staining with Ponceau S (Sigma). Tumour explants were incubated with 10 μg/ml of puromycin for 30 m, before being fixed with 10% neutral buffered formalin (Sigma) and processed for immunohistochemistry.

### Polysome profiling

i) Sucrose density gradient centrifugation and RNA detection

MM cells were treated with 100 μg/ml cycloheximide (CHX) for 3 m prior to harvesting. Cells were lysed in gradient buffer (300 mM NaCl, 15 mM MgCl_2_, 15 mM Tris pH 7.5, 100 μg/ml CHX) plus 1% Triton X-100. Post-nuclear supernatants were layered on 10–50% (w/v) sucrose gradients dissolved in gradient buffer. Gradients were centrifuged at 240,000 × *g* for 2 h at 4 °C in an SW40Ti rotor (Beckman Coulter), then separated through a live optical density 254 nm UV spectrometer (Isco). Gradients were fractionated with continuous monitoring at 254 nm. RNA was isolated from each fraction. First, the volume was adjusted to 2 ml by the addition of H_2_O, followed by the addition of 3 ml of 8 M guanidine HCL. Next, 5 ml of 100% ethanol was added, fractions were cooled to −20 °C and then centrifuged at 24,000 × *g* for 25 min to collect the RNA. The pellets were washed with 75% ethanol, resuspended in 400 ml of TE, pH 7.5, reprecipitated using 0.3 M NaOAC and 2.5 volumes of ethanol. The pellets were washed with 75% ethanol and resuspended in 100 ml of TE, pH 7.5 27,27. RNA was extracted from fractions using Trizol reagent (Invitrogen) according to the manufacturer’s instructions. QuantiTect probes (QIAGEN) for ribosomal proteins mRNAs RPS6, RPS15A, RPL7A, RPL15, RPL24, initiation factors EIF4A1 and EIF4G2 (Dap5), mitochondrial proteins ATP5A1, COX8A, COX7B, UQCRC2 and SDHB, transcript NEO1 and DST, were used according to the manufacturer’s instructions in order to detect the relative mRNA abundance in the gradient fractions. QuantiTect assay (QIAGEN) for ACTB and PABP mRNAs was performed in order to confirm the position subs and polysomes. The polysomal/subpolysomal ratio was determined by Fiji software as the area under the curve.

1. Preparation of fluorescently labelled cDNA for microarray hybridisation
2. Microarrays used were Agilent 8×60k Human Gene Expression arrays (Agilent Technologies LDA UK Ltd.). Equal proportions of RNA from pooled subpolysomal fractions and pooled polysomal fractions were fluorescently labelled, using the Agilent Low Input Quick Amp Labelling Kit, two-colour (Agilent Technologies LDA UK Ltd.). Following hybridisation, the arrays were scanned using an Agilent SureScan High-Resolution scanner and Agilent Feature Extraction software was applied to the resulting images.
3. Array analysis

Raw array data (two-colour, with dye swaps, eight tumour samples and two controls, three repeats) were background corrected using the “normexp” method in the limma R package^68^. The polysome/subpolysome ratios for each probe (M values, log2(red/green)) were compared between the tumour samples and the control samples using RankProd^69^. This is a nonparametric approach that assesses the significance of changes in rank. RP identifies differentially translated probes as separate up- and downregulated lists, and, for each probe, calculates a pfp (percentage of false prediction) which can be used to select significant probes for further study. By way of detailed explanation for this analysis: every gene has a fold change value (FC) between Tumour and Control (see column G in Supplementary Fig. 1). Per every gene, RankProd generates two rankings and two *P* values (see Supplementary Fig. 1). Column K is the position (rank) of the gene in the UP list, column I is the *P* value for the gene for being upregulated. Column S is the gene rank in the Down list, column Q is the *P* value for being downregulated. See, for example, in row 33 of Supplementary Fig. 1, the probe A_23_P329573 for Beta2 Integrin gene ITGB2 (NM_000211). The probe is 6.4 times more intense in Tumour vs Control (Fold Change); the probe is in position 44 in the UP list (see column K) and in position 33265 in the Down list (column S). The probability of Beta2 Integrin gene being in the up list is 2.85E-07 (*P* value in column I), corrected for multiple testing (PFP 0.000227273 in column H).

The probability for Beta2 Integrin gene being downregulated is extremely low (*P* value in column Q and PFP in column P are proximal to 1). Data were considered to be significantly changing if the fold change was at least two at pfp <0.2. This analysis pipeline from raw arrays to lists of significantly changing probes is available as the INCATome R package from CRAN^70^.

### GO term analysis

Lists of significantly up- and downregulated transcripts were uploaded to the online tool bioprofiling.de^71^. Gene Ontology (GO) analysis was performed according to the method described in the online tool. The upregulated genes (list A) and a reference list (list B: all the genes in the genome) are compared. For each GO class f from the set F (all the GOs) the number of genes that have been annotated with f in list A and list B are counted and compared. Then, the null hypothesis H0 (genes that belong to set A are independent of having attribute f) is tested. The output table is provided as Supplementary data 2 (data are expressed in European Number Format). The hierarchical clustering based on Pearson correlation and the heatmap was generated with GeneSpring GX (Agilent).

### UTR analysis

The 5′ UTR, 3′ UTR and CDS sequences for each RefSeq^72^ transcript, for each gene found on the microarray chip, were retrieved from GenBank^73^ records (in-house script). The 5′ UTR sequences were searched for TOP/TOP-like motifs and upstream open-reading frames (uORFs) (in-house scripts). TOP motifs were defined as C[CT]^74^ at the start of the 5′ UTR; if found at positions 2 to 5 from the start of the 5′ UTR they were designated TOP-like^75^. uORFs were defined by an AUG start codon in the 5′ UTR, an in-frame stop codon preceding the end of the main CDS, and a length of at least 9 nt^76^. The 3′ UTR sequences were investigated for the presence of AU-rich elements (in-house script). AU-rich elements were defined as TATTTAT[AT][AT]. The level of structure in each 5′ UTR was calculated using RNAfold from the ViennaRNA Package^77^. The upregulated and downregulated lists of significantly changing genes were compared to the rest of the chip to find any enrichments using *χ*^2^ tests for the presence of TOP/TOP-like motifs, uORFs and AU-rich elements and *t* tests were used for mean 5′ UTR length and 5′ UTR mean free energy. The *t* test carried out on the set of lengths in the upregulated list against the set of lengths in the rest of the array chip to determine whether they came from the same population showed a distinct difference between the pools, with one pool with shorter 5′ UTRs. The list of known TISUs was explored using Table S2 from Sinvani et al.^31^.

### SDS-PAGE and western blotting

Cell lines extracts were generated and subjected to protein quantification by BCA (Thermo Scientific). Protein extracts were subjected to electrophoresis on sodium dodecyl sulphate–polyacrylamide gel electrophoresis followed by transfer to polyvinylidene difluoride membrane (GE Healthcare). Proteins were detected with the relevant antisera using either chemiluminescent reagents (Bio-Rad) or by Odyssey (LI-COR).

Cap m7GTP Sepharose (GE Healthcare) column pull-down was performed on 400 µg of total extracts according to manufacturer instructions. Densitometry was performed with Fiji (Fiji Is Just ImageJ) software according to standard procedure.

### TMA construction and staining

An electronic search of the University Hospitals of Leicester (UHL) NHS Trust histopathology databases was conducted to identify all patients aged 18 years or older who underwent surgery for primary malignant pleural mesothelioma from 2003 to 2014. Surgical procedures included extrapleural pneumonectomy, extended pleurectomy decortication or more localised resections that generated fixed tissue at least 50 mm across or at least five formalin-fixed, paraffin-embedded (FFPE) blocks. All tissue and data used to facilitate the construction of the TMAs were collected under NHS ethics agreement 14/EM/1159 approved by the Northampton committee of the National Research Ethics Service. Owing to the specific HRA exemption for the need for consent for the use of spare archival tissue derived from living patients when samples are handled in an anonymised matter-specific written consent was not required. TMA consist of a recipient formalin-fixed paraffin-embedded block containing 3 × 1 mm donor core donor samples per case, with 40 cases sampled per TMA block. Initially, whole-slide images of haematoxylin and eosin-stained archival mesothelioma were captured at ×40 magnification using the Hamamatsu Nanozoomer XR. Areas containing sufficient tumour material were digitally annotated and corresponding FFPE blocks were retrieved from the diagnostic archive. Where possible, both epithelioid and sarcomatoid regions of tumour differentiation were sampled. A TMA map was produced, 1 mm cores were taken from viable donor blocks and transposed into a pre-prepared recipient TMA block using a semi-automated platform (TMArrayer TM, Pathology Devices). The resulting TMA block was annealed at 37 °C overnight and cooled to room temperature. In all, 4.5 µm sections were prepared from the finished TMA for in situ analysis. Immunohistochemical staining was performed using a chromogenic duplex method, with cytokeratin (MNF) in yellow to aid tissue segmentation and the signal to be quantitated in purple. Assays were performed on a Ventana Discovery Ultra staining platform using the following conditions and counterstained with haematoxylin. Whole-slide images of stained slides were captured at ×40 magnification using the Hamamatsu Nanozoomer XR and analysed using Visiopharm® software. Tumour regions were outlined using a threshold algorithm, and a cytoplasmic H-score was calculated on cells identified using a decision forest algorithm. Cells were placed into categories of 0, 1+, 2+ and 3+ using manually set pixel intensity thresholds corresponding to negative, weak, moderate and strongly stained cells. Automatic H- scores generated by Visiopharm® software were further validated via manual scoring.

### Transmission electron microscopy

Cell pellets were fixed with 2.5% glutaraldehyde (GA) and 2% PFA in the NaHCa buffer (100 mM NaCl, 30 mM HEPES, 2 mM CaCl_2_, adjusted at pH 7.4 with NaOH) for at least 30 min, and then proceeded to the high-pressure freezing and freeze substitution (Leica EM HPM100 and AFS2, Leica Microsystems, Vienna Austria). In brief, the specimens were incubated in 0.1% tannic acid in anhydrous acetone at −90 °C for at least 24 h, washed with pure acetone at −90 °C and substituted in 1% osmium tetroxide, 0.1% uranyl acetate, and 1% pure water in acetone, with temperature-controlled under −90 °C for 72 h, at −60 °C for 8 h, at −30 °C for 8 h (with an interval of 30 °C/hr). After several washing with pure acetone at 4 °C, samples were embedded in Epon and Araldite mixture (TAAB Laboratories Equipment Ltd., Reading UK). After the polymerisation at 65 °C for few days, the ultrathin-sections (∼60 nm) obtained by Ultramicrotome (Leica Ultracut UCT, Vienna, Austria) were mounted in EM grids, stained with lead citrate and then observed by 200 kV transmission electron microscope (FEI Talos, Oregon USA) with Ceta-16M CMOS-based camera (4kx4k pixels under 16 bit dynamic range). For the conventional procedure, the cells were fixed in the GA/PFA mixture and post-fixed with 0.25% osmium tetroxide/0.25% potassium ferrocyanide and 1% tannic acid. After being stained in a bloc with 5% aqueous uranyl acetate, dehydration with a series of ethanol and infiltration were completed for the plastic embedding in TER (TAAB epoxy resin). A shape descriptor on Fiji software (https://imagej.net/Fiji) was used to quantify mitochondrial parameters (area, minor axis, major axis, circularity) in the micrographs. Elongated mitochondrial particles are considered when the major axis/minor axis ratio is higher than 3. Enlarged mitochondrial particles displayed a degree of circularity > 0.8 and area > 0.5 μm^2^.

### Immunofluorescence and confocal microscopy

MpM primary cells and untransformed controls cells were grown on a glass coverslip in a six-well culture plate. Fixation was performed with 3% PFA (Sigma), 2% sucrose in PBS 1× solution for 10 m at 25 °C. Permeabilization was performed in Hepes-TritonX100 (20 mM HEPES pH 7.4, 300 mM sucrose, 50 nM NaCl, 3 mM MgCl_2_, 0.5% TritonX100) for 5 m at 25 °C. After blocking in 2% bovine serum albumin (BSA), cells were incubated with anti-mitochondrial protein ATPB for 30 m at 37 °C, washed in 0.2% BSA in PBS, and incubated with proper Alexa Fluor conjugated secondary antibody (Invitrogen) for 30 m at 37 °C. Nuclei were stained with 4′,6-diamidino-2-phenylindole (Sigma). Confocal images were taken at the equatorial plane of the nucleus with LSM510 META (Zeiss) equipped with ×63 immersion lens. Images were acquired with Zen2009 software (Zeiss). The mitochondria network morphology (elongated, mixed or enlarged/fragmented) was determined per every observed cell, on at least three fields per condition.

### Metabolic change measurement

Rates of oxidative phosphorylation were determined by measuring the rate of OCR using a Seahorse XF96 extracellular flux (XF) analyser (Seahorse Bioscience, Billerica, MA, USA). Primary MpM or control cell lines were seeded in an XF96 cell culture microplate at 4 × 10^4^ cells/well 24 h before the assay and incubated at 37 °C under 5% CO_2_ in a humidified atmosphere. In all, 24 h prior to assay, 1 ml of Seahorse calibrant was added to each well of a Seahorse XF96 utility plate and the probes on the sensor cartridge were allowed to soak at 37 °C under CO_2_-free conditions. 1 h prior to assay, cells were washed three times in unbuffered bicarbonate and serum-free Dulbecco’s Modified Eagle Medium (pH 7.4) containing 1 mM sodium pyruvate,11 mM glucose and 2 mM glutamax; then covered in media, and incubated for 1 h at 37 °C under CO_2_-free conditions. The OCR was measured with the Seahorse XF Cell Mito Stress Test Kit (Agilent) following the manufacturer instructions.

### LC-MS analysis

Samples were analysed on a Q Exactive Plus Orbitrap mass spectrometer (Thermo Scientific, Waltham, MA, USA) coupled with a Thermo Ultimate 3000 HPLC system. Sample extracts (5 µl) were injected and metabolites were separated on a ZIC-pHILIC column (SeQuant, 150 × 2.1 mm, 5 µm, Merck KGaA, Darmstadt, Germany), equipped with a ZIC-pHILIC guard column (SeQuant, 20 × 2.1 mm). The total analysis time was 25 m and mobile phase acetonitrile content was decreased from 80% (20% 20 mM ammonium carbonate, pH 9.2) to 20% acetonitrile over a 15 m gradient (flow rate: 200 μL/min; column temperature: 45 °C). Ions across a mass range of 75–1000 m/z were collected using the Q Exactive Plus mass spectrometer operating at a resolution of 70,000 (at 200 m/z), with electrospray (ESI) ionisation and polarity switching. A pooled sample (mixture of all sample extracts) was prepared and analysed multiple times throughout acquisition as a QC control. The same pooled sample was analysed separately in positive and negative single ionisation mode using data-dependent fragmentation (ddMS2) for metabolite identification. Data were acquired with Thermo Xcalibur software. Data analysis and statistical tests (analysis of variance; Benjamini–Hochberg) were performed using Compound Discoverer software (Thermo Scientific v3.1). Retention times were aligned across all sample data files (maximum shift 2 min, mass tolerance 5 ppm). Unknown compound detection (minimum peak intensity 5e5) and grouping of compound adducts were carried out across all samples (mass tolerance 5 ppm, RT tolerance 0.7 min). Missing values were filled using the software’s Fill Gap feature (mass tolerance 5 ppm, S/N tolerance 1.5). Metabolite identification was achieved by matching the mass and retention time of observed peaks to an in-house database generated using metabolite standards (mass tolerance 5 ppm, RT tolerance 0.5 min), Peak annotations were confirmed using mzCloud (ddMS2) database search (precursor and fragment mass tolerance of 10 ppm, match factor threshold 50) and searching predicted compositions (mass tolerance 5 ppm, minimum spectral fit and pattern coverage of 30% and 90%, respectively) against the HMDB database. Based on data collected on the same system for commercial standards, further analysis of metabolites was performed using Thermo TraceFinder v4.1 software. Intracellular metabolites were normalised to the average number of cells collected from three wells that were experimentally identical to the ones used for metabolite extraction. The statistical analysis of the data showing the metabolic effects of Torc1 and Torc2 inhibition was performed applying a paired *t* test with Benjamini–Hochberg correction to log2 transformed and centred data using R. The outputs are shown in supplementary data 3 and 4.

### Stable isotope labelling using ^13^C6-glucose, ^13^C5-glutamine and ^13^C4-aspartate

Cells were seeded in triplicate in six-well plates at 50,000 cells per well (7 T and 8 T) or 100,000 cells per well (NMS) in 2 ml of RPMI (supplemented with l-glutamine (2 mM), hEGF (20 ng/ml), hydrocortisone (1 μg/ml) and 10% FBS). After 24 and 48 h, 2 ml of the culture medium was added. After 72 h, the culture medium was changed to 7 ml supplemented RPMI containing 150 μM ^13^C4 l-aspartic acid, 2 mM ^13^C5 l-glutamine, 11.11 mM ^13^C6 d-glucose (Sigma) or without tracer. Cells were extracted after 24 h in 400 μl of ice-cold extraction solution, and samples were analysed by LC/MS as described above.

### Collection of clinical specimens for ex vivo samples

Human investigations were performed after Research Ethics Committee approval (LREC 08/H0406/226). Resected mesothelioma specimens were collected with the patient written consent. All patients underwent surgery for radical decortication without prior chemotherapy or radiotherapy. Solid tumours were immediately immersed in ice-cold RPMI-1640 supplemented with penicillin (100 U/ml), streptomycin (100 μg/ml) and 10% FBS. Samples were transported to the laboratory for primary cell culturing within 1 h of collection. Tumours were processed in order to obtain small tumour blocks (~1–3 mm in diameter) and then transferred into a six-well plate (eight blocks per well) in the above-mentioned medium at 37 °C and 5% CO_2_. Blocks were treated with 100 nM Torin1, 100 nM Hippuristanol, 100 nM AZD2014 or 250 nM AZD8186 for 72 h and then fixed in 10% neutral buffered formalin (Sigma). Immunohistochemical staining for Ki67, pospho-Ser240/244 rpS6, puromycin (12D10), ATP5A and SDH were performed using DAB (3,3′- Diaminobenzidine); haematoxylin was used as counterstain. Cytokeratin (MNF) was used to identify tumour areas within tumour blocks. Quantification of Ki67 positive cells and quantifications of pospho-Ser240-244 rpS6, puromycin, ATP5A and SDH staining intensities within tumour area was performed by using Fiji software or H-scores were generated automatically by Visiopharm® software.

### Animal experimentation

All experiments involving mice were approved by the local animal welfare committee and conducted under UK Home Office licence, PE47BC0BF (DJM, Glasgow) and were compliant with the ARRIVE guidelines (https://www.nc3rs.org.uk/arrive-guidelines). Mice were maintained on a constant 12 hr light/dark cycle under controlled climate (19–22 °C; 45–65% humidity), fed and watered ad libitum and all were mixed background (FVBN and C57Bl/6). Mice bearing the combination of target alleles *Cdkn2a*^*−/−*^*;Nf2*^*fl/fl*^*;Tp53*^*fl/fl*^ were generously provided by Anton Berns, Netherlands Cancer Institute (NKI)^55^. Genotyping was performed by Transnetyx Inc. Tumour initiation was induced in young adult mice (aged 8–10 weeks) and mice were maintained until predefined humane clinical endpoints were reached. Lentivirus expressing Cre recombinase was purchased from the University of Iowa vector core facility. Long fibre amosite asbestos was generously provided by Rodger Duffin (MRC Inflammation Unit, Edinburgh). Intrapleural injection of asbestos was carried out by injection directly into the pleural space without perforating the lungs by the addition of a sleeve over the tip of the 27-Gauge needle; this prevented the needle from passing through the pleural space into the lungs^20^. AZ8186 and AZ2014 were supplied by Astra Zeneca under MTA/RCA with the CRUK Beatson Institute. Drugs were dissolved and administered in 0.5% HPMC, 0.1% Tween 20, H2O vehicle. Both males and females were included on all treatment and control arms. AZ8186 and AZ2014 were supplied by Astra Zeneca under MTA/RCA with the CRUK Beatson Institute. Drugs were dissolved and administered in 0.5% HPMC, 0.1% Tween 20, H2O vehicle. Endpoint monitoring was performed by CRUK Beatson Institute Biological Services facility staff without knowledge of genotype.

For histological analysis, mouse tissues were fixed with 10% neutral buffered formalin overnight. All immunohistochemistry was performed as described^78^ on 4 µm formalin-fixed paraffin-embedded sections which had previously been heated to 60 °C for 2 h. Peroxidase blocking was performed for 10 mins in 1% H_2_O_2_ diluted in H_2_O, followed by heat-mediated antigen retrieval. Non-specific antibody binding was blocked with 3% BSA or 5% normal goat serum for 1 h at RT. The following antibodies were used at the indicated dilution and indicated antigen retrieval method: phospho-S6 (Cell Signaling Tech. 5634, 1;1000), ER2 antigen retrieval solution (Leica). Anti-rabbit Ig (Vector Labs) was used as the secondary antibody. The HRP signal was detected using liquid DAB (Invitrogen). Sections were counterstained with haematoxylin and cover-slipped using DPX mount (Cellpath, UK).

### Reporting summary

Further information on research design is available in the Nature Research Reporting Summary linked to this article.

## Supplementary information

Supplementary Information

Description of Additional Supplementary Files

Supplementary Data 1

Supplementary Data 2

Supplementary Data 3

Supplementary Data 4

Reporting Summary

## Data Availability

The polysome data generated in this study have been deposited in https://www.ebi.ac.uk/arrayexpress/experiments with accession code E-MTAB-8167 [ArrayExpress - E-MTAB-6351]. The processed data from this study of the mRNAs whose polysome association changes can be found in the excel spreadsheets in Supplementary data 1 and the GO analysis is present in Supplementary data 2. The raw patient data collected under NHS ethics agreement 14/EM/1159 are protected and are not available owing to data privacy laws. However, anonymised raw patient data are available upon application to Professor John Le Quesne (Professor in Molecular Pathology Institute of Cancer Sciences, John.LeQuesne@glasgow.ac.uk). The metabolite data used to generate Figs. 4 and 6 are available as excel spreadsheets in Supplementary data 3 and 4. Raw metabolomics data are available upon request to Dr. David Sumpton (Head of Metabolomics, Beatson Institute for Cancer Research, UK) via email (d.sumpton@beatson.gla.ac.uk). Source data are provided with this paper.

## Source data

Source Data
