# Supplementary material for: The pathogenesis of mesothelioma is driven by a dysregulated translatome
Source: Nat Commun. 2021 Aug 13;12:4920. doi: 10.1038/s41467-021-25173-7 (PMC8363647; doi:10.1038/s41467-021-25173-7)
Supplement: Supplementary file 1 — Supplementary Information [file 41467_2021_25173_MOESM1_ESM.pdf]

a

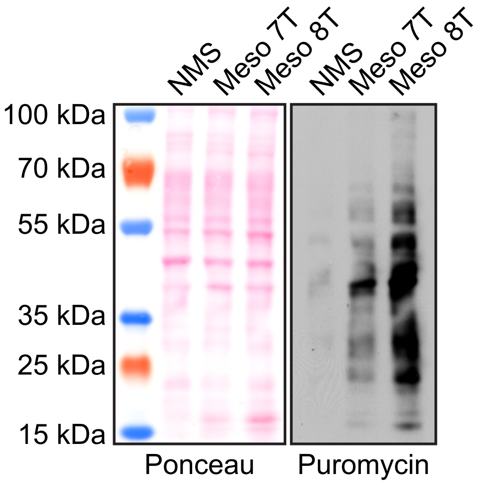

b

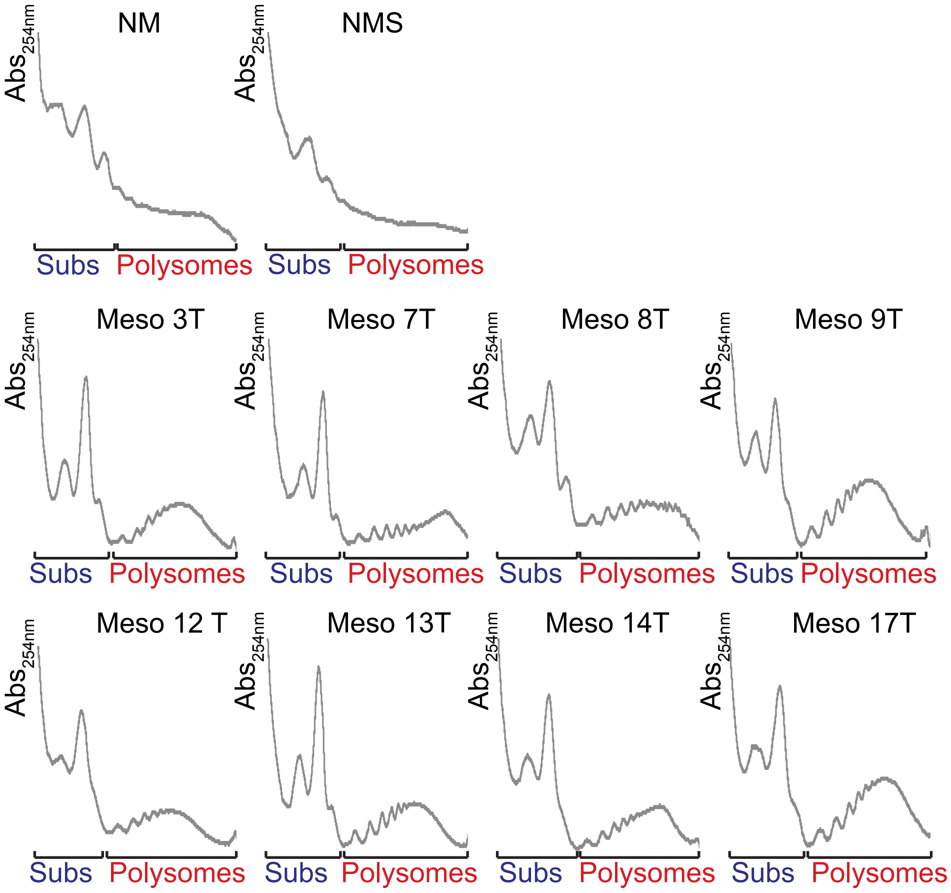

c

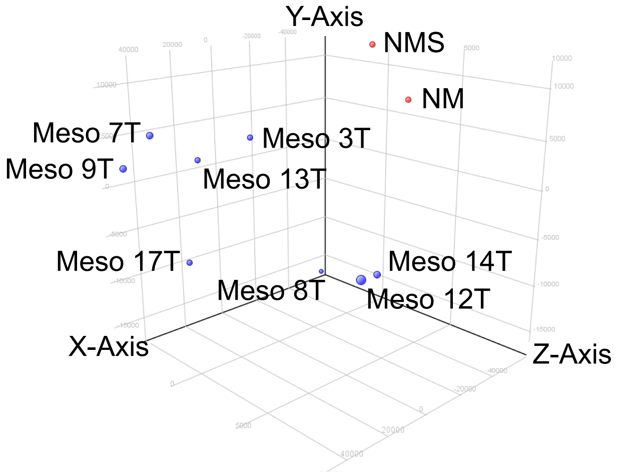

Supplementary Figure 1

Supplementary Figure 1: Primary MpM cell lines have increased translation rate as compared to the control untransformed cells

A) MpM cell lines Meso 7T and Meso 8T and untransformed mesothelium control (NMS) were pulsed with 10 μg/ml of puromycin for 10 minutes, before being lysed. Extracts were separated by SDS-PAGE and stained with red Ponceau as a loading control. Antibody against puromycin shows increase translation in MpM samples. This experiment was repeated on three occasions

B) Polysome profiles of either healthy mesothelial cells (NM and NMS) or 8 MpM derived cell lines were performed. MpM cell lines have higher polysomes as compared to the control.

C) PCA clustering of control cells (NM and NMS) or 8 MpM primary lines.

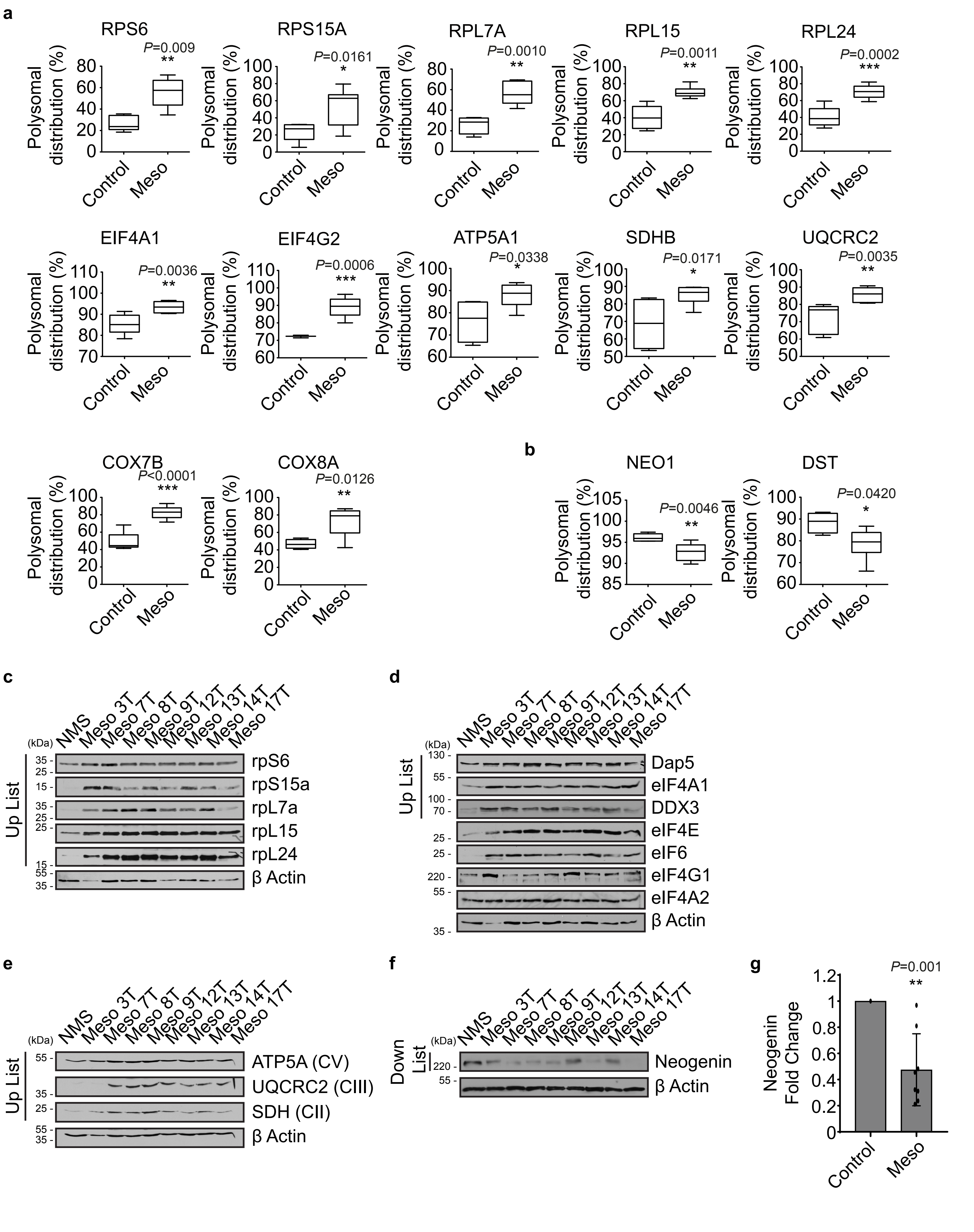

Supplementary Figure 2

Supplementary Figure 2: Primary MpM cell lines specifically translate ribosomal proteins, protein synthesis machinery and mitochondrial components

A) qPCR validations of mRNAs extracted from polysomes and from sub-polysomes. Probes (QuantiTect) for ribosomal proteins mRNAs RPS6, RPS15A, RPL7A, RPL15, RPL24, initiation factors EIF4A1 and EIF4G2 (Dap5), mitochondrial proteins ATP5A1, COX8A, COX7B, UQCRC2 and SDHB were used to detect the relative mRNA abundance between polysomal and sub- polysomal fractions. Error bars represent standard deviation and the measure of centre of the error bars is the mean (n=3, where n=number of independent biological repeats). Significance was assessed using two sided unpaired student's t-test with multiple comparisons, p values are shown on the bar graphs.

B) qPCR validation of mRNA extracted from polysomes and from sub-polysomes. Probes (QuantiTect) for NEO1 and DST were used to detect the relative abundance of the RNA between polysomal and sub-polysomal fractions. Error bars represent standard deviation and the measure of centre of the error bars is the mean (n=3, where n=number of independent biological repeats). Significance was assessed using two sided unpaired student's t-test with multiple comparisons, p values are shown on the bar graphs. NEO1 and DST1 are less polysomally associated in MpM cells.

C) Western blot analysis of 8 primary cell lines derived from patients with MpM and from normal mesothelium (NMS), which were probed with antibodies against rpsS6, rps15a, rpL7a, rpL15, rpL24. Actin was used as a sample integrity control. Source data are provided as a Source Data file. These experiments were repeated on three occasions with similar results.

D) Western blot analysis of 8 primary cell lines derived from patients with MpM and from normal mesothelium (NMS), which were probed with antibodies against Dap5, eIF4A1, DDX3, eIF4E, eIF6, eIF4G1, eIF4A2. The same Actin blots shown in Figure 1C was used as a sample integrity control, since the same cell extracts used for these experiments. These experiments were repeated on three occasions with similar results. Source data are provided as a Source Data file.

E) Western blot analysis of 8 primary cell lines derived from patients with MpM and from normal mesothelium (NMS), which were probed with antibodies against ATP5A, UQCRC2, SDH, VDAC. Actin as a sample integrity control. These experiments were repeated on three occasions with similar results. Source data are provided as a Source

F) Western blot analysis of 8 MpM cell lines and normal mesothelium (NMS) which were probed with antibodies against Neogenin. Actin was used as a sample integrity control. Source data are provided as a Source Data file.

G) Densitometric analysis of the western blots in Supplementary Figure. Protein levels in MpM-derived cell lines are compared to NMS control cells. Error bars represent standard deviation and the centre of the error bars represents the mean (n=8 biological repeats) and significance was assessed using unpaired two sided Student's t-test adjusted for multiple comparisons. Source data are provided as a Source Data file.

F) Densitometric analysis of the western blots in Supplementary Figure 2F, Protein levels in MpM-derived cell lines are compared to NMS control cells. Error bars represent standard deviation with the centre of the bar representing the mean (n=8 biological repeats) and significance was assessed using a two sided unpaired Student's t-test adjusted for multiple comparisons.

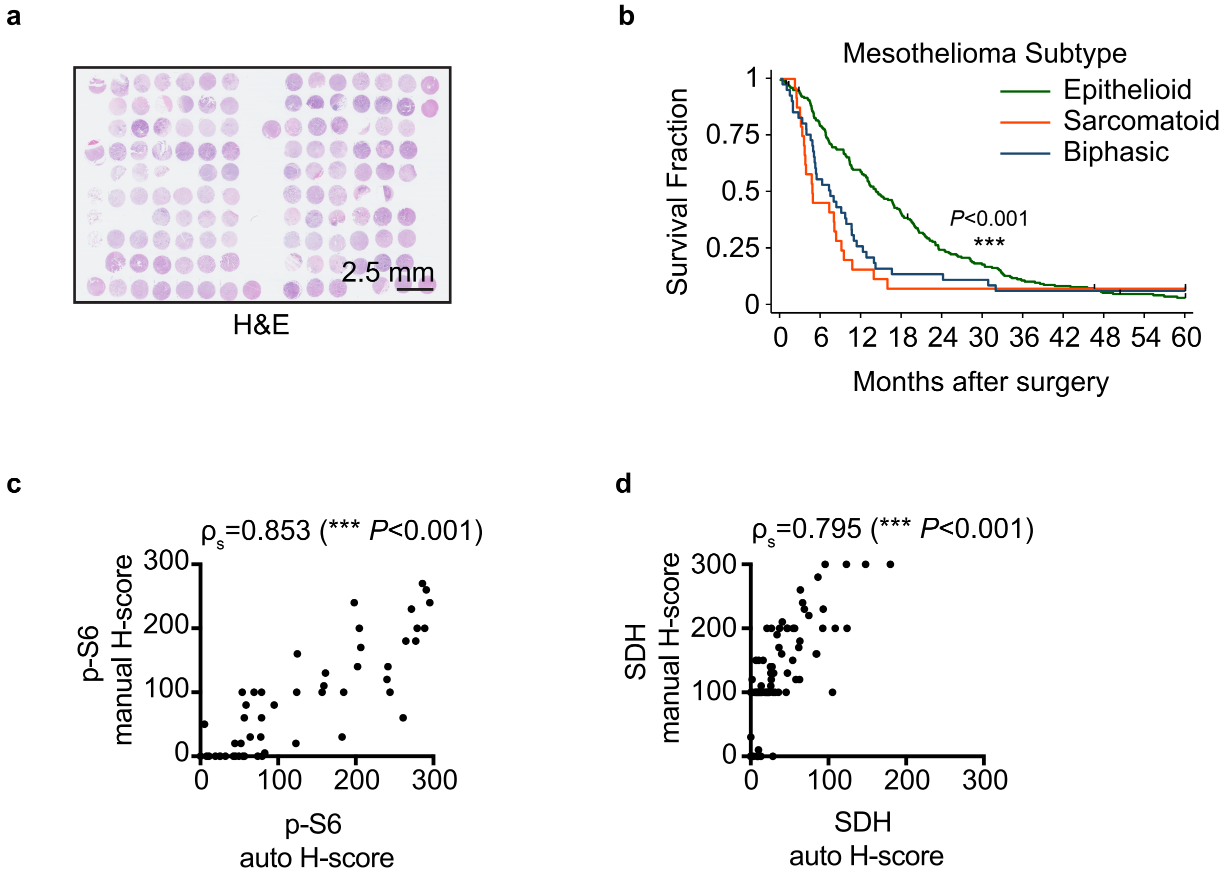

Supplementary Figure 3

Supplementary Figure 3: Mesothelioma tissue micro array shows that histological sub-type predicts survival

A) Representative picture of haematoxylin and eosin stain (H&E) of a tissue micro array (TMA).

B) Kaplan-Meier plot, showing MpM histological sub-type is a strong predictor of survival. Of the 272 patients included in the TMAs, 205 were diagnosed with epithelioid MpM, 24 with sarcomatoid MpM, and 43 with biphasic MpM. 270 of these were included for survival analysis - 2 patients who died on date of surgery were excluded (one epithelioid MpM and one biphasic MpM). In the first 5 years following surgery (60 months), there were 192 deaths in the epithelioid MpM group, 22 deaths in the sarcomatoid MpM group, and 38 deaths in the biphasic MpM group. A log-rank test was performed to determine the significance of differences in survival by diagnosed mesothelioma subtype - this was highly significant (** p=0.025). In agreement with previous studies (reviewed in (5)), in our patient series histological sub-type is a strong predictor of survival with a significantly worse outcome for patients with sarcomatoid compared to epithelioid MpM.

C) Spearman r correlation between manually calculated H-scores and H-scores generated automatically by Visiopharm® software for TMA stained with P rpS6 antibody (*** p=0.001).

D) Spearman r correlation between manually calculated H-scores and H-scores generated automatically by Visiopharm® software for TMA stained with SDH antibody (*** p=0.001).

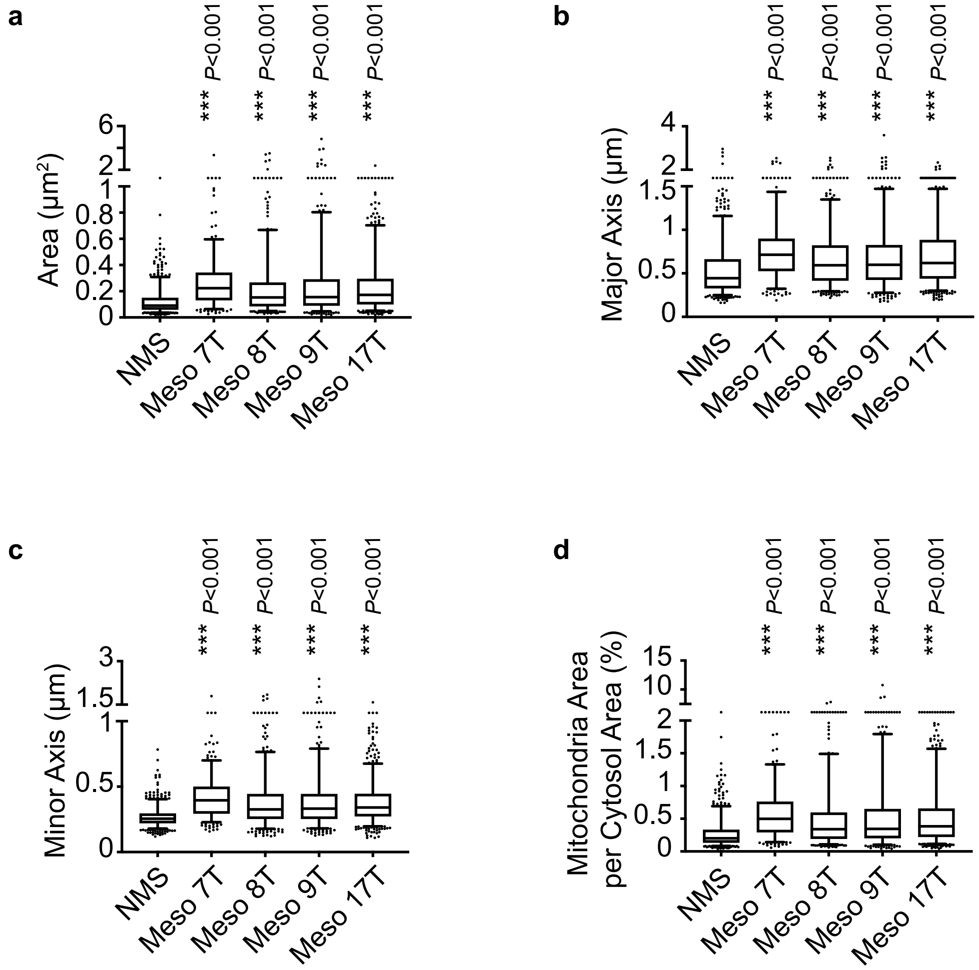

Supplementary Figure 4

Supplementary Figure 4: Mitochondrial morphology is altered in Primary MpM cell lines In total n=369 mitochondrial particles from 20 electron micrographs of primary MpM cell lines (Meso 7T, Meso 8T, Meso 9T and Meso17T) and a control (NMS) from Figure 3A were analysed. The area (A), the major (B) and the minor axis (C), and the percentage of the area covered (D) are presented in the box-plots including the 5-95 percentiles. Significance was assessed using Mann Whitney test. In each case the box plots show the box from the first quartile to the third quartile. The middle line represents the median. The whiskers go up to the maximum and minimum values. P values are shown on the graphs.

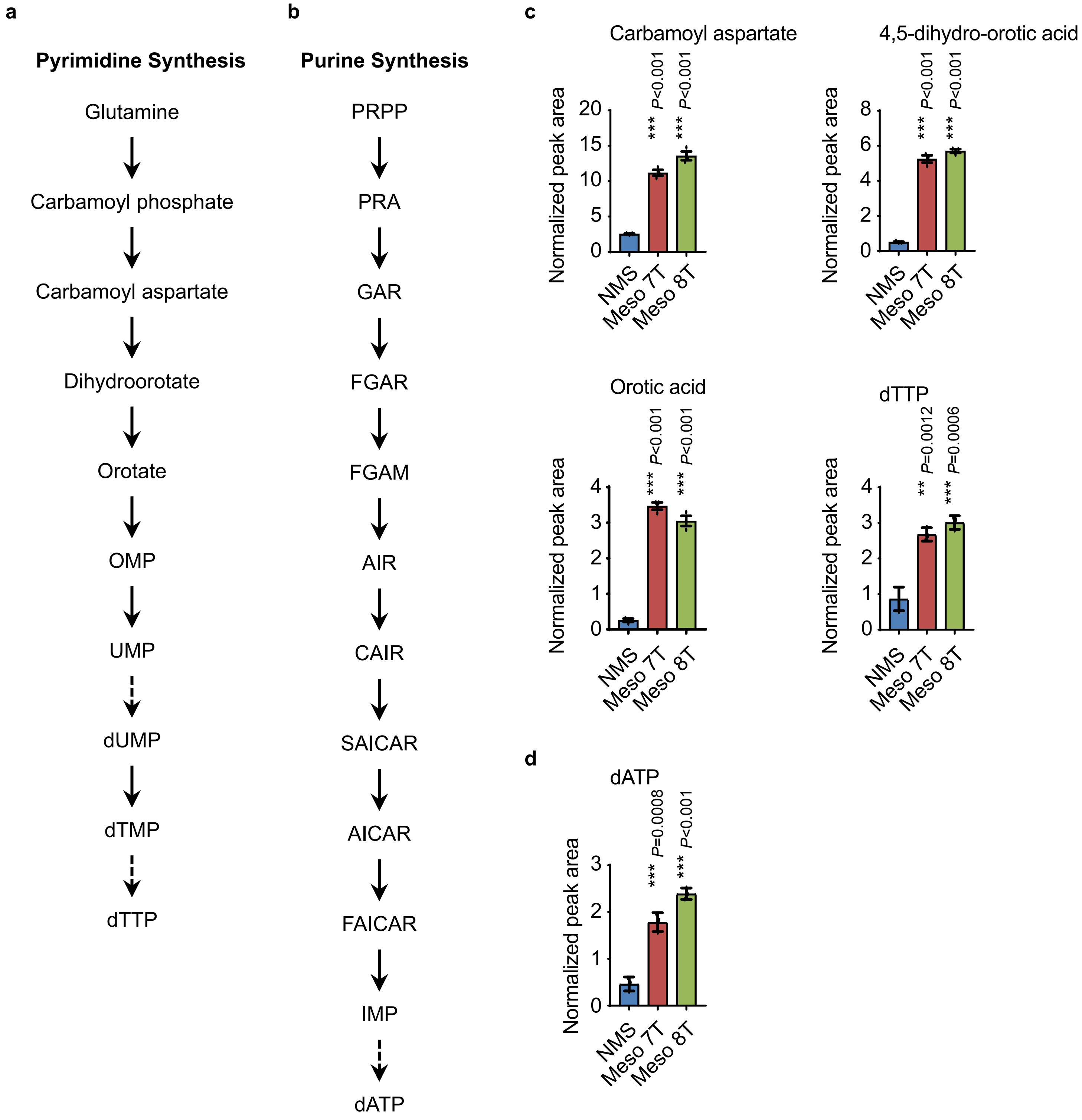

Supplementary Figure 5

Supplementary Figure 5: Metabolic changes in MpM derived cell lines

A) Schematic to show the Pyrimidine Synthesis pathway.

B) Schematic to show the Purine Synthesis pathway.

C) Intracellular abundance of Pyrimidine Synthesis intermediates: carbamoyl aspartate, dihydroorotate, orotate, dTTP. Data represent the mean ± SD. Significance was assessed using unpaired two sided student's t-test adjusted for multiple comparisons

D) Intracellular abundance of dATP. Data represent the mean ± SD. Significance was assessed using unpaired two sided student's t-test adjusted for multiple comparisons.

Metabolic data in C and D are from three independent biological experiments, each with 3 technical replicates.

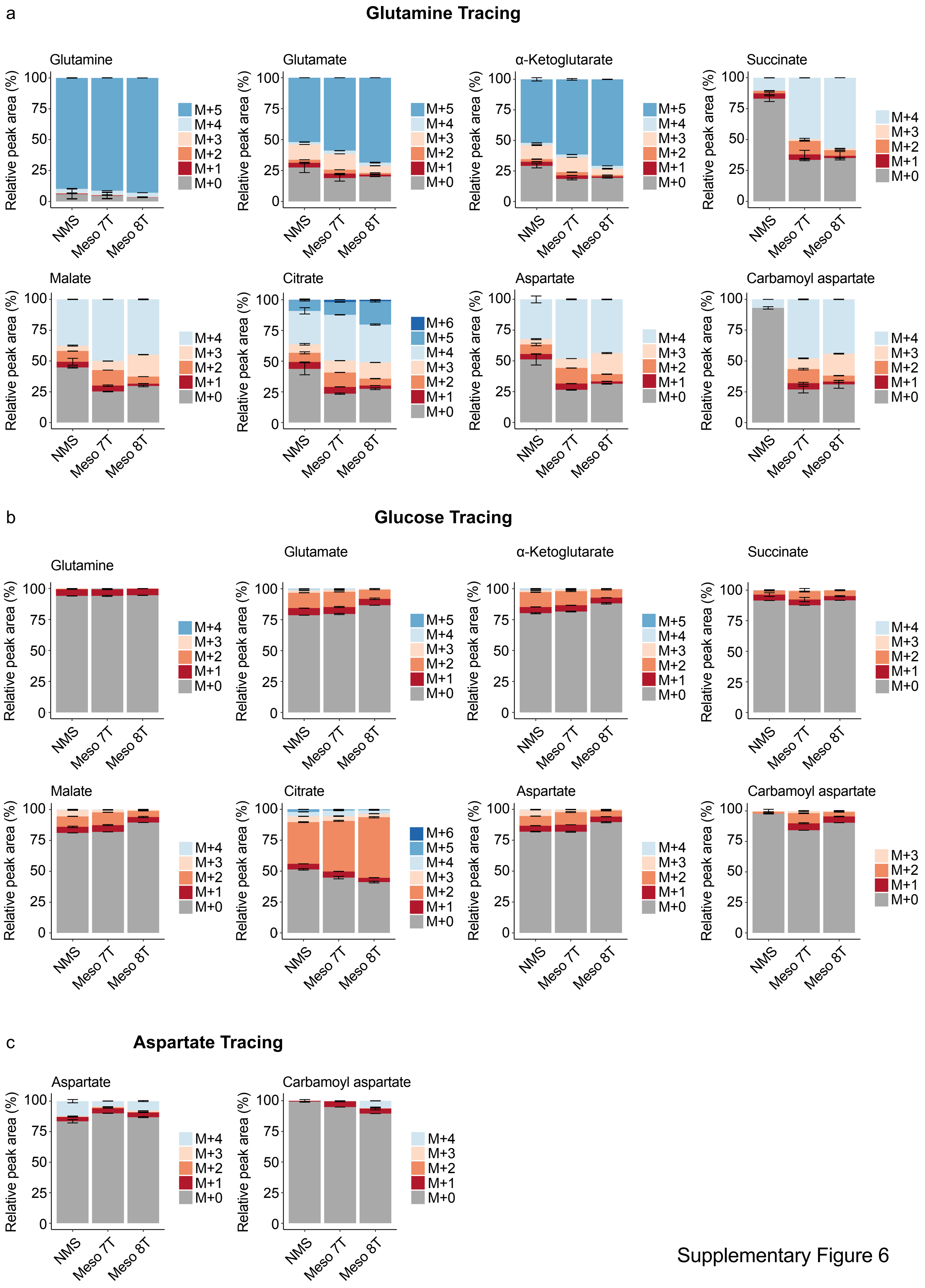

Supplementary Figure 6

Supplementary Figure 6: Metabolic state in MpM derived cell lines and NMS cells.

A) Intracellular relative abundance of metabolites in cells cultured with $^{13}C_5$ L-glutamine.

Data represent the mean ± SD obtained from an experiment with 3 technical replicates.

B) As in A, but cells were cultured with $^{13}C_6$ D-glucose.

C) As in A, but cells were cultured with $^{13}C_4$ L-aspartic acid.

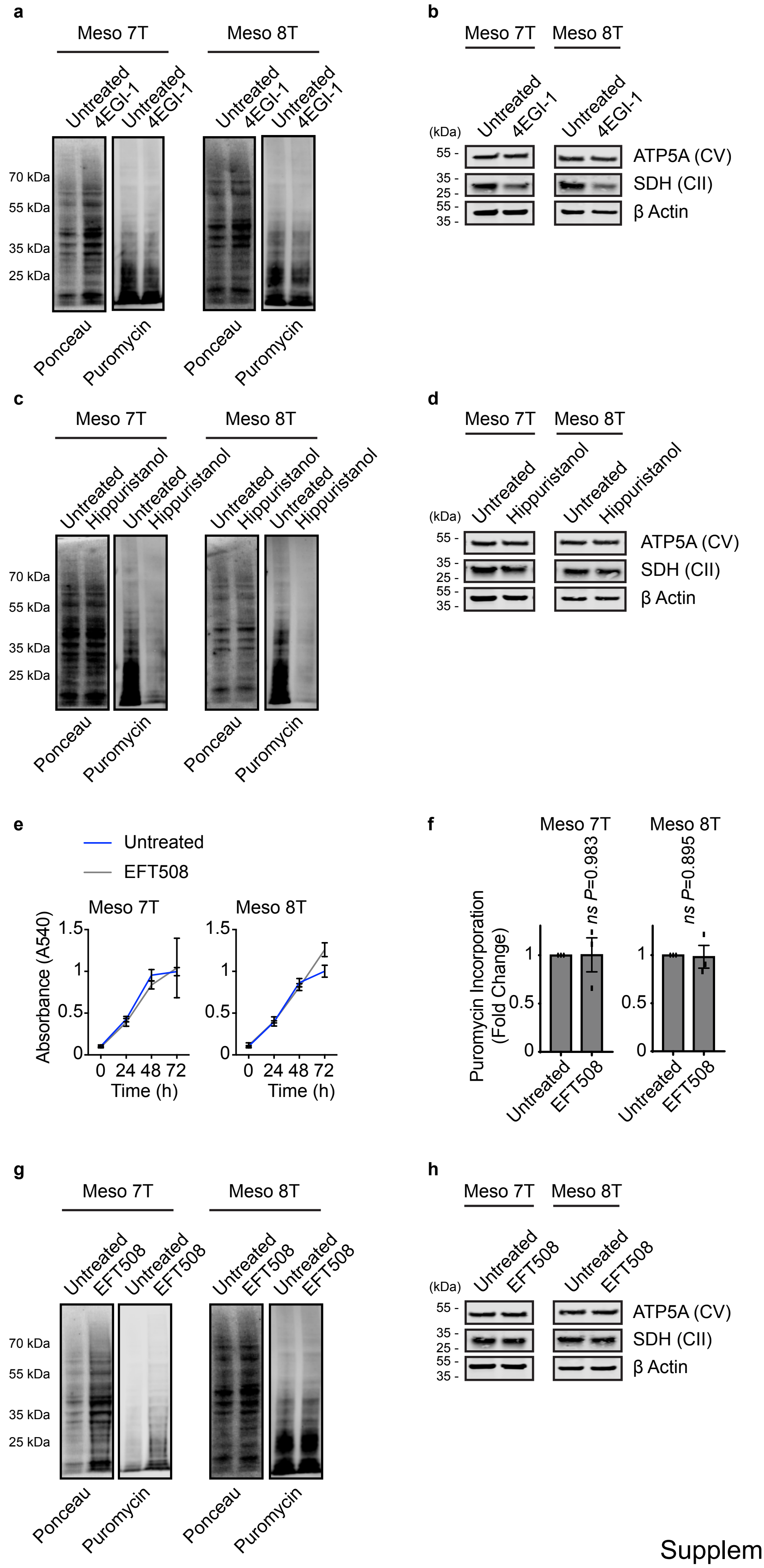

Supplementary Figure 7

Supplementary Figure 7: Inhibition of cap complex formation, reduces translation rate and mitochondria protein levels

A) MpM cell lines Meso 7T and Meso 8T treated with 25 µM 4EGI-1 or left untreated, were pulsed with 10 µg/ml of puromycin for 10 minutes, before being lysed. Extracts were separated by SDS-PAGE and stained with red Ponceau as a loading control. Antibody against puromycin shows decrease of translation in samples treated with 4EGI-1. These experiments were repeated on three independent occasions with similar results obtained.

B) MpM cell lines Meso 7T and Meso 8T treated with 25 µM 4EGI-1 or left untreated. Extracts were separated by SDS-PAGE. Membranes were probed with antibodies to assess changes in mitochondria proteins. Actin was used as a sample integrity control. These experiments were repeated on three occasions with similar results obtained. Source data are provided as a Source Data file

C) MpM cell lines Meso 7T and Meso 8T treated with 100 nM Hippuristanol or left untreated, were pulsed with 10 µg/ml of puromycin for 10 minutes, before being lysed. These experiments were repeated on three occasions with similar results obtained.

D) MpM cell lines Meso 7T and Meso 8T treated with 100 nM Hippuristanol or left untreated. Extracts were separated by SDS-PAGE. Membranes were probed with antibodies to assess changes in mitochondria proteins. These experiments were repeated on three occasions with similar results obtained. Actin was used as a sample integrity control. Source data are provided as a Source Data file

E) Extracts were separated by SDS-PAGE. Membranes were probed with antibodies to assess changes in mitochondrial proteins. Actin was used as a sample integrity control. These experiments were repeated on three occasions with similar results obtained. Primary MpM cells (Meso 7T or Meso 8T) were treated with 10 nM EFT508 and the rate of cell growth was assessed over a 72-hour time period with a crystal violet assay. Error bars represent standard deviation and centre of the bar represents the mean (n=4).

F) Primary MpM cells (Meso 7T or Meso 8T) were treated with 10 nM EFT508. Puromycin incorporation from n=3 where n= number of independent biologicals repeats of the puromycin incorporation experiment (representative in Supplementary Figure 7G). Error bars represent standard deviation error bars represent the mean. No significance was assessed using a two sided unpaired Student's t-test.

H) MpM cell lines Meso 7T and Meso 8T treated with 10 nM EFT508 or left untreated, were pulsed with 10 µg/ml of puromycin for 10 minutes, before being lysed. Extracts were separated by SDS-PAGE and stained with red Ponceau as a loading control. Antibody against puromycin shows no change of translation in samples treated with EFT508.

MpM cell lines Meso 7T and Meso 8T treated with 10 nM EFT508 or left untreated. Extracts were separated by SDS-PAGE. Membranes were probed with antibodies to assess changes in mitochondria proteins. Actin was used as a sample integrity control. These experiments were repeated on three occasions with similar results obtained. Source data are provided as a Source Data file

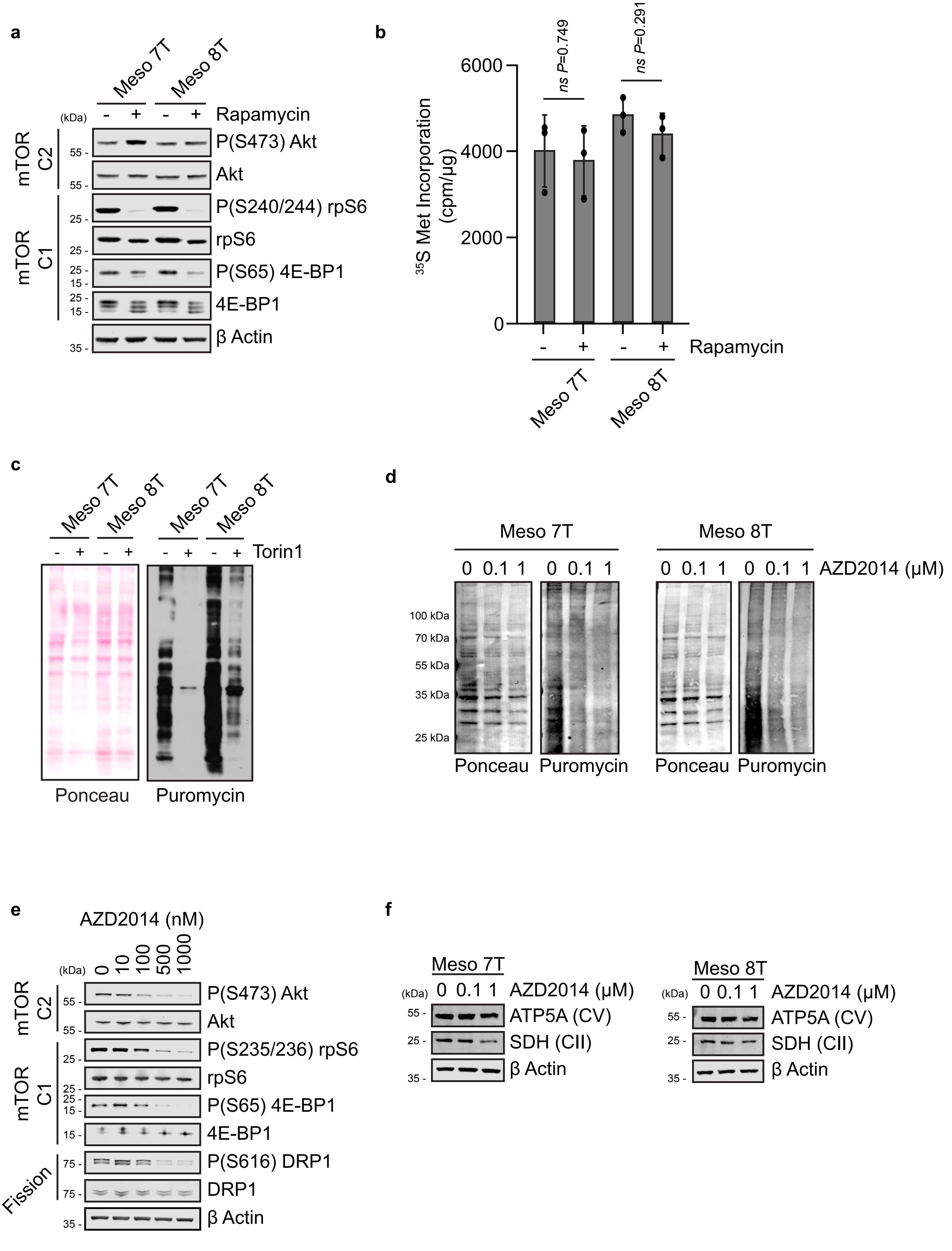

Supplementary Figure 8

Supplementary Figure 8: Inhibition of mTORC1 and 2 reduces the translation rate

A) Western blot analysis of primary MpM cell lines (Meso 7T and Meso 8T) treated with 100 nM Rapamycin for 30 minutes, or left untreated and probed with antibodies to assess changes in mTOR pathway. Actin was used as a sample integrity control. These experiments were repeated on three independent occasions with similar results obtained. Source data are provided as a Source Data file

B) Primary MpM derived cell lines Meso 7T and Meso 8T were incubated with 100 nM Rapamycin for 30 minutes, or left untreated, followed by 33 µCi 35S methionine and the rate of incorporation over a 30 minutes period was assessed. Error bars represent standard deviation and the centre of the bar represents the mean (n=3, where n= number of independent biological repeats of the methionine incorporation experiment). Significance was assessed using a two sided unpaired student t-test adjusted for multiple comparisons. **C)** MpM cell lines Meso 7T and Meso 8T treated with 100 nM Torin 1 for 30 minutes or left untreated, were pulsed with 10 µg/ml of puromycin for 10 minutes, before being lysed. Extracts were separated by SDS-PAGE and stained with red Ponceau as a loading control. Antibody against puromycin shows decrease of translation in samples treated with Torin 1.

These experiments were repeated on three independent occasions with similar results obtained. **D)** MpM cell lines Meso 7T and Meso 8T treated with increasing dose of AZD2014 as depicted in the figure, were pulsed with 10 µg/ml of puromycin for 10 minutes, before being lysed. Extracts were separated by SDS-PAGE and stained with red Ponceau as a loading control. Antibody against puromycin shows decrease of translation in samples treated with AZD2014. These experiments were repeated on three independent occasions with similar results obtained.

E) Western blot analysis of Meso 8T treated with increasing dose of AZD2014 as depicted in the figure. Membranes were probed with antibodies shown to assess changes in mTOR pathway and mitochondria fission. These experiments were repeated on three independent occasions with similar results obtained.Actin was used as a sample integrity control. Source data are provided as a Source Data file

F) Western blot analysis of primary MpM cell lines (Meso 7T and Meso 8T) treated with increasing dose of AZD2014 as shown and harvested after 24 hours. Membranes were probed with antibodies to assess changes in mitochondria proteins. Actin was used as a sample integrity control. These experiments were repeated on three independent occasions with similar results obtained. Source data are provided as a Source Data file.

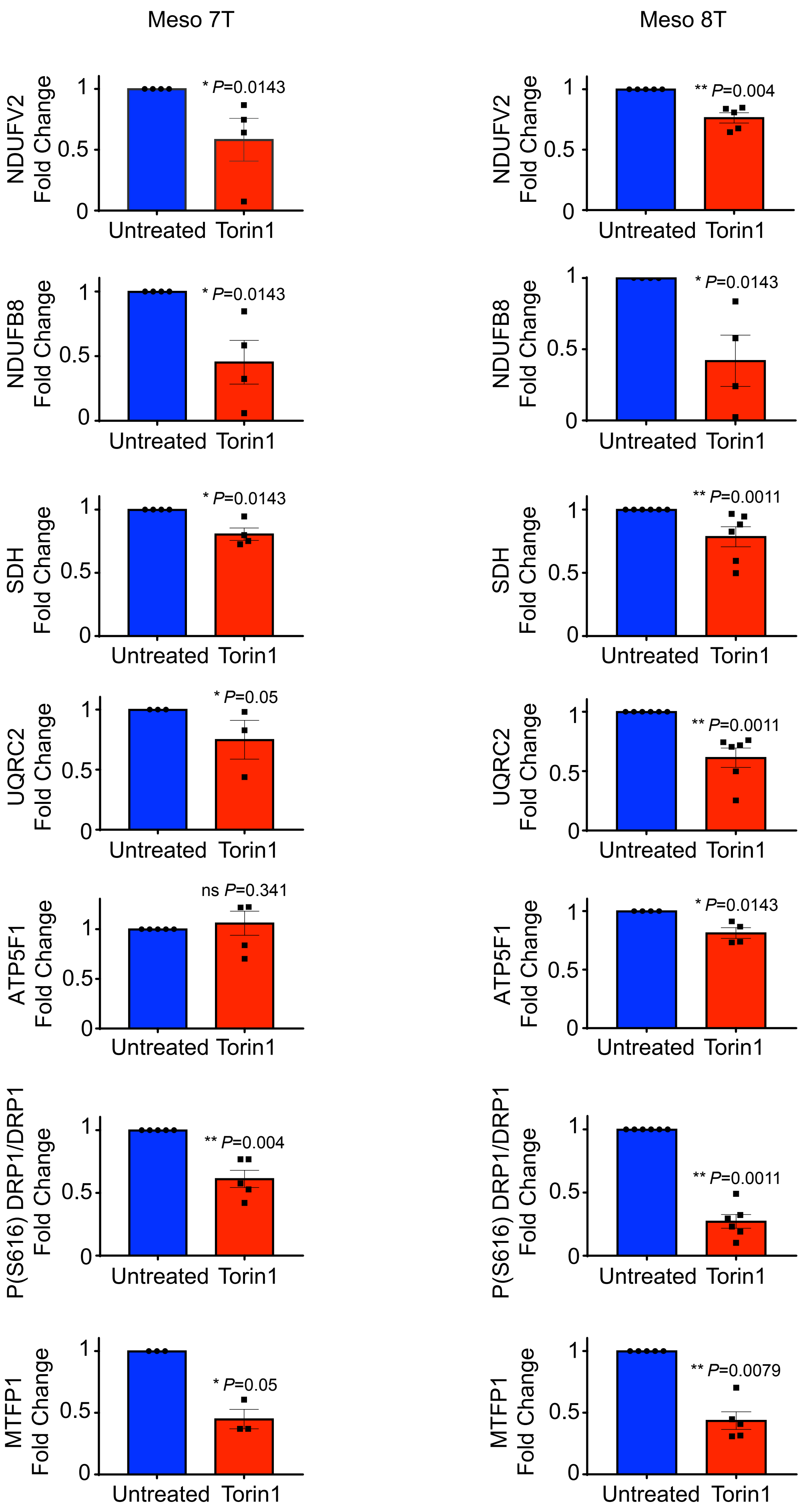

Supplementary Figure 9

Supplementary Figure 9: Densitometry analysis of western blots related to Figure 5K

Densitometry analysis of western blot analysis (Figure 5K) of primary MpM cell lines (Meso 7T and Meso 8T) treated with 100 nM Torin 1 for 24 hours and probed with antibodies to assess changes in mTOR pathway, mitochondrial protein expression and mitochondrial dynamics. Actin was used as a sample integrity control. Mann Witney significance test was performed (n=3 independent biological repeats). Error bars show standard error of the mean, centre of the error bars is the mean.

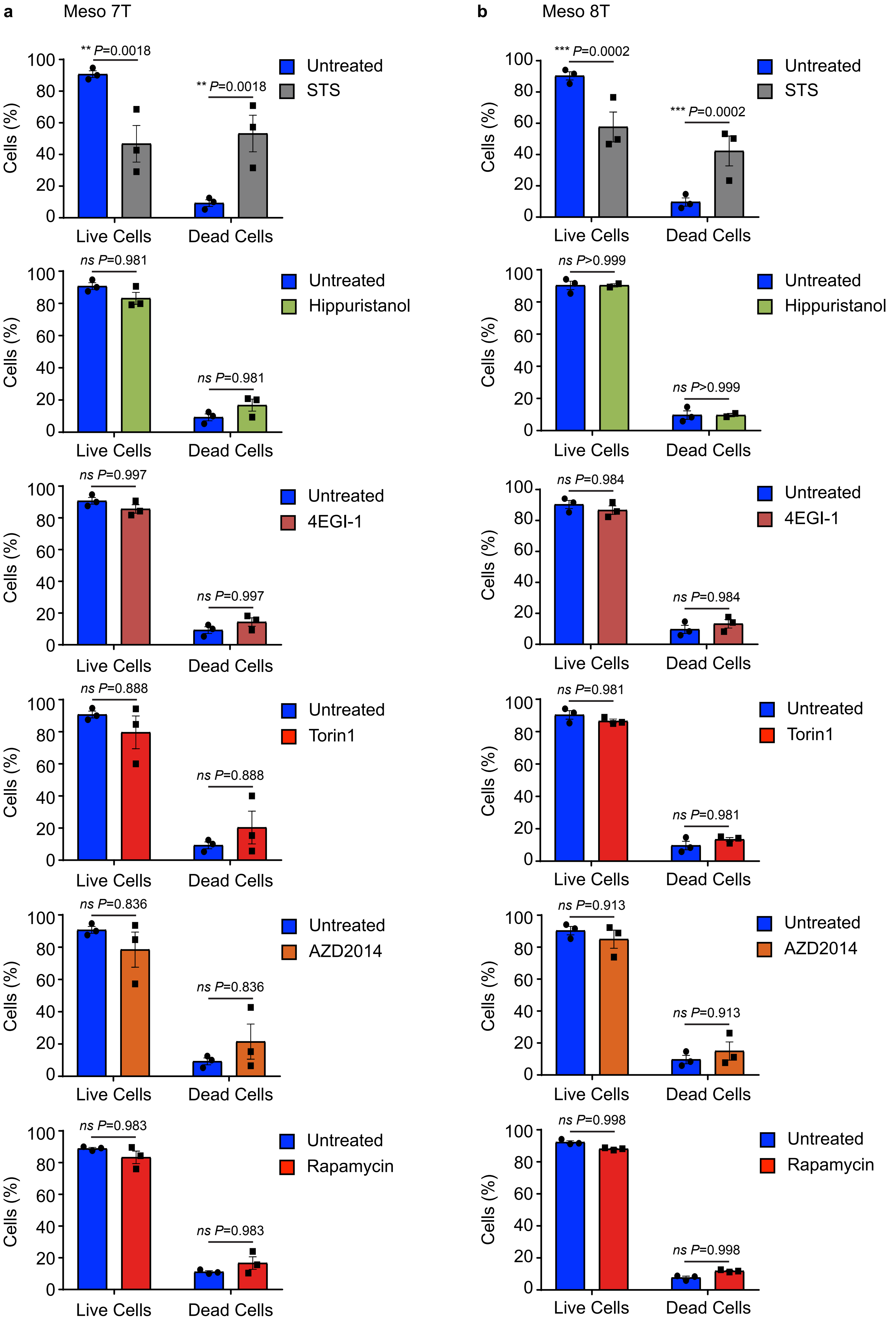

Supplementary Figure 10

Supplementary Figure 10: Translation inhibition does not induce apoptosis in primary mesaothelioma cell lines

A and B) FACS analysis of primary MpM cell lines Meso 7T (A) and Meso 8T (B) treated with 100nM Hippuristanol, 25μM 4EGI-1, 100nM Torin1, 100nM AZD 2014 and 100nM Rapamycin respectively for 48h. 5 μM Staurosporine (STS) for 24h was used as a positive control. Cell death was assesed using Annexin/Draq7 co-staining. A 2way ANOVA test was performed (n=3 independent biological repeats). Error bars represent standard error of the mean, centre of the error bars is the mean.

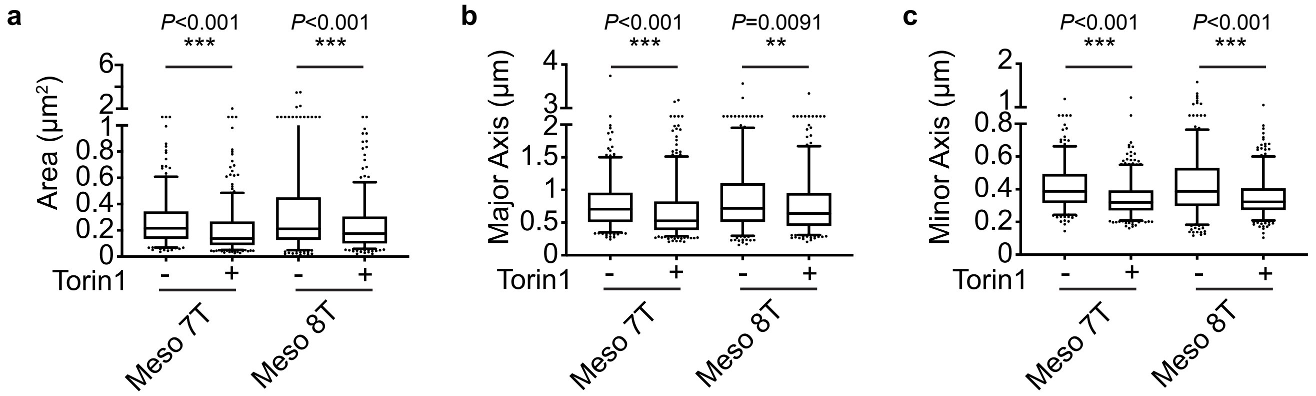
a
P<0.001
***
P<0.001
***
Area (µm2)
6
2
1
0.8
0.6
0.4
0.2
0
Torin1
-
+
-
+
Meso 7T
Meso 8T
b
P<0.001
***
P=0.0091
**
Major Axis (µm)
4
3
2
1.5
1
0.5
0
c
P<0.001
***
P<0.001
***
Minor Axis (µm)
2
1
0.8
0.6
0.4
0.2
0

Supplementary Figure 11: Inhibition of mTORC1 and C2 restores mitochondria morphology Approximately 300-400 mitochondrial particles from 20 electron micrographs of primary MpM cell lines (Meso 7T and Meso 8T) treated with 100 nM Torin 1 or left untreated from Figure 6G were analysed. The area, the major and the minor axis are presented in the box-plots including the 5- 95 percentile. The box plots show the box from the first quartile to the third quartile. The middle line represents the median. The whiskers go up to the maximum and minimum values. Significance was assessed using Mann Whitney test. P values are shown on the graphs.

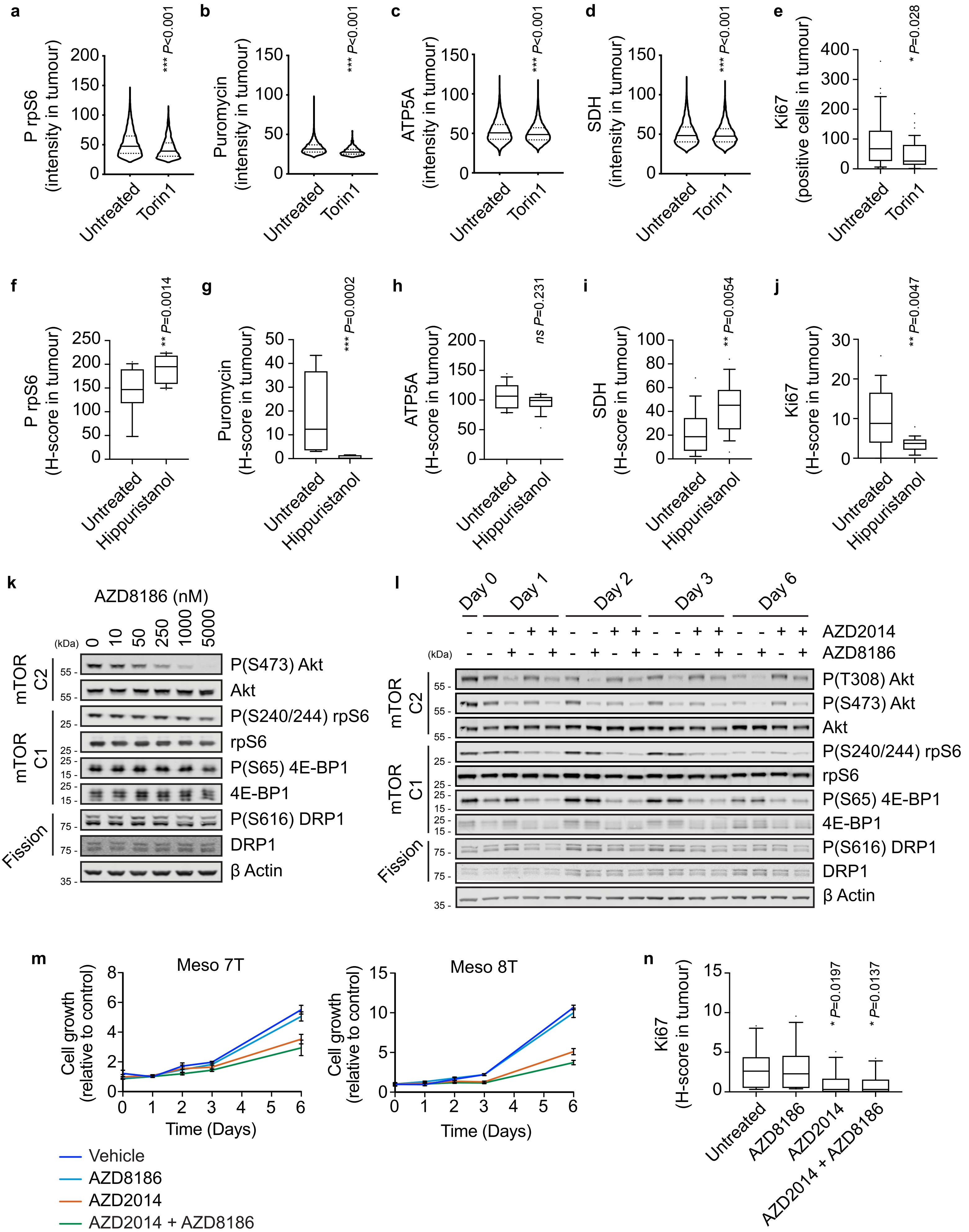

Supplementary Figure 12

Supplementary Figure 12: Translation inactivation inhibits tumour cell growth

A) Mesothelioma explants were cultured for 72h with Torin 1 or with vehicle. Quantification of the intensity of the staining of P rpS6 within tumour areas is performed. The distribution of the intensities is shown as a violin plot. The black line shows the median, the dotted lines show the quartiles of the distribution. Mann Witney significance test was performed (***p<0.001, three patients, n=8 explants each).

B) Mesothelioma explants were cultured for 72h with Torin 1 or with vehicle, then pulsed with 10 μg/ml of puromycin for 30 minutes, before being fixed. Quantification of the intensity of the staining of Puromycin within tumour areas is performed. The distribution of the intensities is shown as a violin plot. The black line shows the median, the dotted lines show the quartiles of the distribution. Mann Witney significance test was performed (***p<0.001, three patients, n=8 explants each).

C) Mesothelioma explants were cultured for 72h with Torin 1 or with vehicle. Quantification of the intensity of the staining of ATP5A within tumour areas is performed. The distribution of the intensities is shown as a violin plot. The black line shows the median, the dotted lines show the quartiles of the distribution. Mann Witney significance test was performed (***p<0.001, three patients, n=8 explants each) the variant plot is based on 20,000 data points.

D) Mesothelioma explants were cultured for 72h with Torin 1 or with vehicle. Quantification of the intensity of the staining of SDH within tumour areas is performed. The distribution of the intensities is shown as a violin plot. The black line shows the median, the dotted lines show the quartiles of the distribution. Mann Witney significance test was performed (***p<0.001, three patients, n=8 explants each).

E) Mesothelioma explants were cultured for 72h in presence or absence of Torin1. Quantifications of the cells positive for Ki67 staining within tumour areas is shown in the box-plots including the 10-90 percentiles. Mann Witney significance test was performed (three patients, n=8 explants each). P=0.028 The box plots show the box from the first quartile to

the third quartile. The middle line represents the median. The whiskers go up to the maximum and minimum values

F) Mesothelioma explants were cultured for 72h in presence or absence of Hippuristanol. H-score quantification of rpS6 phosphorylation (mTOR activity) within tumour was performed by Visiopharm® software. Mann Witney significance test was performed (n=16 explants, p=0.014). The box plots show the box from the first quartile to the third quartile. The middle line represents the median. The whiskers go up to the maximum and minimum values

G) Mesothelioma explants were cultured for 72h in presence or absence of Hippuristanol, then pulsed with 10 □g/ml of puromycin for 30 minutes, before being fixed. H-score quantification of Puromycin within tumour was performed by Visiopharm® software. Mann Witney significance test was performed (n=8 explants, p=0.0002). The box plots show the box from the first quartile to the third quartile. The middle line represents the median. The whiskers go up to the maximum and minimum values

H) Mesothelioma explants were cultured for 72h in presence or absence of Hippuristanol. H-score quantification of ATP5A within tumour was performed by Visiopharm® software. Mann Witney significance test was performed (n=16 explants, ns). The box plots show the box from the first quartile to the third quartile. The middle line represents the median. The whiskers go up to the maximum and minimum values

I) Mesothelioma explants were cultured for 72h in presence or absence of Hippuristanol. H-score quantification of SDH within tumour was performed by Visiopharm® software. Mann Witney significance test was performed (n=16 explants,p=0.0054). The box plots show the box from the first quartile to the third quartile. The middle line represents the median. The whiskers go up to the maximum and minimum values

J) Mesothelioma explants were cultured for 72h in presence or absence of Hippuristanol. H-score quantification of Ki67 staining within tumour was performed by Visiopharm® software. Mann Witney significance test was performed (n=16 explants, p=0.0047).

The box plots show the box from the first quartile to the third quartile. The middle line represents the median. The whiskers go up to the maximum and minimum values

K) Western blot analysis of primary MpM cells Meso 8T treated with increasing dose of AZD8186 as depicted in the figure. Membranes were probed with antibodies to assess changes in mTOR pathway and mitochondria fission. Actin was used as a sample integrity control. These experiments were repeated on three independent occasions with similar results obtained. Source data are provided as a Source Data file.

L) Western blot analysis of Meso 8T cells treated with either vehicle, or 100 nM AZD2014, or 250 nM AZD8186 or with the combination of the two compounds, over a 6-day time period as depicted in the figure. Membranes were probed with antibodies to assess changes in mTOR pathway and mitochondria fission. Actin was used as a sample integrity control. These experiments were repeated on three independent occasions with similar results obtained. Source data are provided as a Source Data file.

M) Primary MpM cells (Meso 7T or Meso 8T) were treated with either vehicle, or 100 nM AZD2014, or 250 nM AZD8186 or with the combination of the two compounds and the rate of cell growth was assessed over a 6-day time period with a crystal violet assay. Error bars represent standard deviation, centre of the bar represents the mean (n=6, n=number of independent biological repeats of the growth rate experiment).

N) Mesothelioma explants were cultured for 72h with either vehicle, or 100 nM AZD2014, or 250 nM AZD8186 or with the combination of the two compounds. H-score quantification of Ki67 staining within tumour was performed by Visiopharm® software. Mann Witney significance test was performed (two patients, n=16 explants each, P=0.0197 for AZD2014 and p=0.0137 for AZD2014+AZD8186). The box plots show the box from the first quartile to the third quartile. The middle line represents the median. The whiskers go up to the maximum and minimum values

a

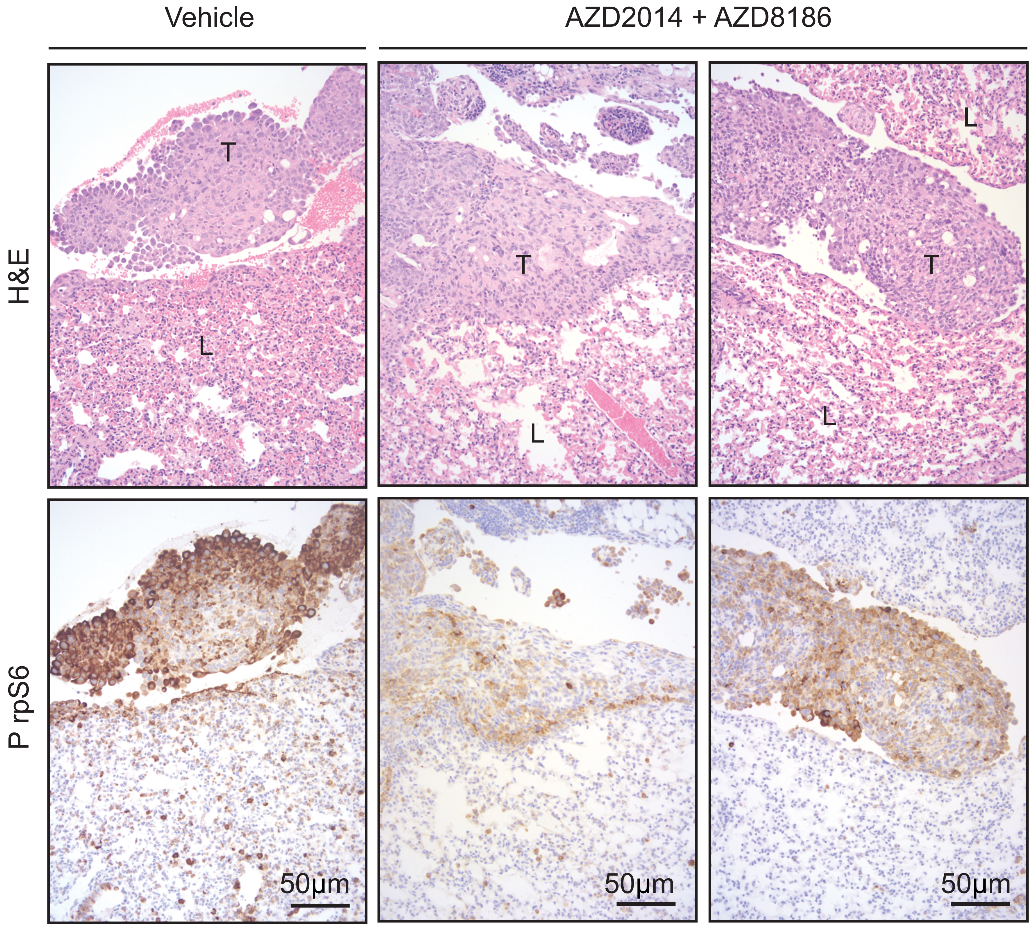

b

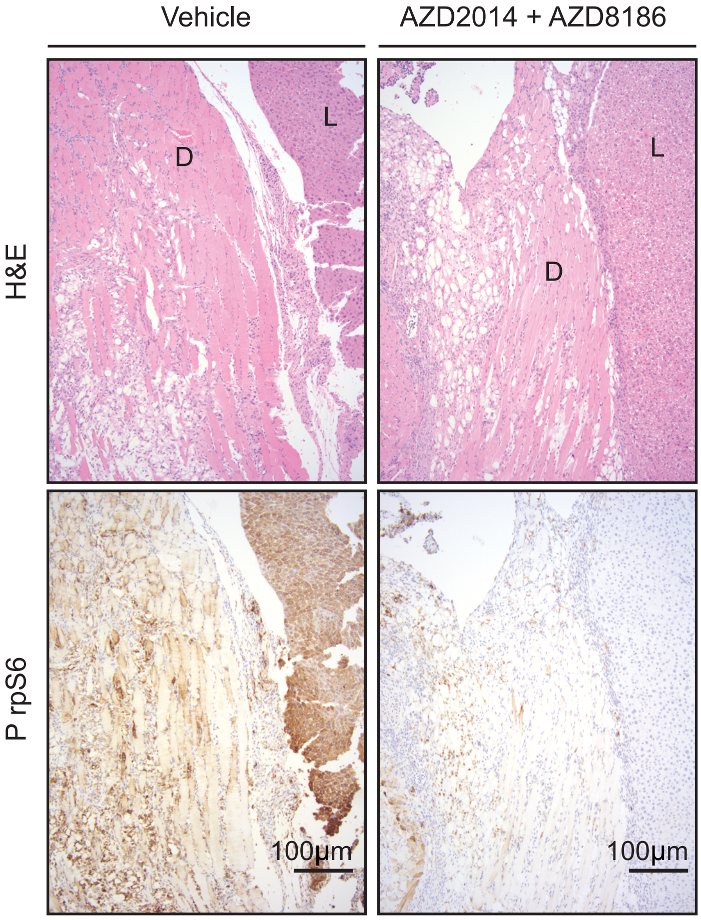

Supplementary Figure 13

Supplementary Figure 13: mTORC 1 and 2 inactivation inhibits tumour cell growth in mouse models of MpM

A) Haematoxylin and eosin stain (H&E) and IHC analysis of P rpS6 in the mice tumour (T) and lung (L). Similar stains were obtained from three independent tumours

B) Haematoxylin and eosin stain (H&E) and IHC analysis of P rpS6 in the mice liver (L) and diaphragm (D). Similar staining pattern was obtained from three independent tumours.

Supplementary Data

Supplementary Data 1

This contains the lists of the translationally up and down regulated mRNAs.

Supplementary Data

This contains the GO analysis from file 1

Supplementary Data 3

This file contains the metabolomic data used to generate Figure 4

Supplementary Data 4

This file contains the metabolomic data used to generate Figure 6
