# Supplementary material for: The pathogenesis of mesothelioma is driven by a dysregulated translatome
Source: Nat Commun. 2021 Aug 13;12:4920. doi: 10.1038/s41467-021-25173-7 (PMC8363647; doi:10.1038/s41467-021-25173-7)
Supplement: Supplementary file 2 — Description of Additional Supplementary Files [file 41467_2021_25173_MOESM2_ESM.pdf]

Description of Additional Supplementary Files

File Name: Supplementary Data 1
Description: This contains the lists of the translationally up and down regulated mRNAs.

File Name: Supplementary Data 2
Description: This contains the GO analysis from file 1

File Name: Supplementary Data 3
Description: This file contains the metabolomic data used to generate Figure 4

File Name: Supplementary Data 4
Description: This file contains the metabolomic data used to generate Figure 6
