# Supplementary material for: The pathogenesis of mesothelioma is driven by a dysregulated translatome
Source: Nat Commun. 2021 Aug 13;12:4920. doi: 10.1038/s41467-021-25173-7 (PMC8363647; doi:10.1038/s41467-021-25173-7)
Supplement: Supplementary file 7 — Reporting Summary [file 41467_2021_25173_MOESM7_ESM.pdf]

nature portfolio

Corresponding author(s): Anne Willis

Last updated by author(s): 16-07-2021

Reporting Summary

Nature Portfolio wishes to improve the reproducibility of the work that we publish. This form provides structure for consistency and transparency in reporting. For further information on Nature Portfolio policies, see our Editorial Policies and the Editorial Policy Checklist.

Statistics

For all statistical analyses, confirm that the following items are present in the figure legend, table legend, main text, or Methods section.

n/a	Confirmed	
☐	☒	The exact sample size (n) for each experimental group/condition, given as a discrete number and unit of measurement
☐	☒	A statement on whether measurements were taken from distinct samples or whether the same sample was measured repeatedly
☐	☒	The statistical test(s) used AND whether they are one- or two-sided<br>*Only common tests should be described solely by name; describe more complex techniques in the Methods section.*
☐	☒	A description of all covariates tested
☐	☒	A description of any assumptions or corrections, such as tests of normality and adjustment for multiple comparisons
☐	☒	A full description of the statistical parameters including central tendency (e.g. means) or other basic estimates (e.g. regression coefficient) AND variation (e.g. standard deviation) or associated estimates of uncertainty (e.g. confidence intervals)
☐	☒	For null hypothesis testing, the test statistic (e.g. F, t, r) with confidence intervals, effect sizes, degrees of freedom and P value noted<br>*Give P values as exact values whenever suitable.*
☒	☐	For Bayesian analysis, information on the choice of priors and Markov chain Monte Carlo settings
☒	☐	For hierarchical and complex designs, identification of the appropriate level for tests and full reporting of outcomes
☐	☒	Estimates of effect sizes (e.g. Cohen's d, Pearson's r), indicating how they were calculated

Our web collection on statistics for biologists contains articles on many of the points above.

Software and code

Policy information about availability of computer code

Data collection	ViennaRNA = v2.1.8, Visopharm version 2020.08.4.9377
Data analysis	Raw array data (two-colour, with dye swaps, eight tumour samples and two controls, three repeats) were background corrected using the 'normexp' method in the limma R package The polysome/subpolysome ratios for each probe (M values, log2(red/green)) were compared between the tumour samples and the control samples using RankProd. This method detects differentially expressed genes under two experimental conditions and those were considered to be significantly changing if the fold change was at least two at pfp < 0.2. This analysis pipeline from raw arrays to lists of significantly changing probes is now available as the INCATome R package from CRAN.<br>Limma = 3.18.13; RankProd = 2.34; GenBank = Release 209;

For manuscripts utilizing custom algorithms or software that are central to the research but not yet described in published literature, software must be made available to editors and reviewers. We strongly encourage code deposition in a community repository (e.g. GitHub). See the Nature Portfolio guidelines for submitting code & software for further information.

Data

Policy information about availability of data

All manuscripts must include a data availability statement. This statement should provide the following information, where applicable:
- Accession codes, unique identifiers, or web links for publicly available datasets
- A description of any restrictions on data availability
- For clinical datasets or third party data, please ensure that the statement adheres to our policy

The polysome data generated in this study have been deposited in https://www.ebi.ac.uk/arrayexpress/experiments with accession code E-MTAB-8167 [ArrayExpress - E-MTAB-6351]. The processed data from this study of the

mRNAs whose polysome association changes can be found in the excel spread sheets in Supplementary data 1 and the GO analysis is present in Supplementary data 2. The raw patient data collected under NHS ethics agreement 14/EM/1159 are protected and are not available due to data privacy laws. However, anonymised raw patient data are available upon application to Professor John Le Quesne (Professor in Molecular Pathology Institute of Cancer Sciences, John.LeQuesne@glasgow.ac.uk). The metabolite data used to generate Figures 4 and 6 are available as excel spread sheets in Supplementary data 3 and 4. Raw metabolomics data are available upon request to Dr. David Sumpton (Head of Metabolomics, Beatson Institute for Cancer Research, UK) via email (d.sumpton@beatson.gla.ac.uk). Source data are provided with this paper which contain the original scans for the western blots shown in Figure 3E, 5B, 5C, 5K and 5L and Supplementary Figures, 2, 7, 8A, 8E, 8F and 12.

Field-specific reporting

Please select the one below that is the best fit for your research. If you are not sure, read the appropriate sections before making your selection.

☒ Life sciences ☐ Behavioural & social sciences ☐ Ecological, evolutionary & environmental sciences

For a reference copy of the document with all sections, see nature.com/documents/nr-reporting-summary-flat.pdf

Life sciences study design

All studies must disclose on these points even when the disclosure is negative.

Sample size	1. The polysome profiling was carried out on 8 biological repeats and 3 technical repeats in each case. We chose to use 8 cell lines derived from individuals with mesothelioma since our earlier research on polysome profiling from diffuse large B-cell lymphoma (doi: 10.1038/leu.2013.295) has shown that this number of biological repeats provides very reproducible biological data and is sufficient to take into account tumour cell heterogeneity. The controls were normal mesothelial cells collected from 6 individuals. There will not be a large individual variation in the polysome profiles from the healthy control cells, and therefore biological repeats from 6 individual was sufficient in this case. 2. The data for the human mesothelioma tissue arrays was taken from 270 individuals (we only had access to 270 patient samples and no sample size calculations were carried out). The tissue explants were carried out using from 3 individual patient tumours and 8 different samples from each tumour (no sample size calculations were carried out, as we only had access to 3 patient tumours, the data obtained were significant). Overall, we analysed from 20,000 individual data points.<br>3. The seahorse analysis was carried out on 6 independent occasions (our prior experiments using this apparatus provided us with the information that this number of repeats is sufficient to take into account experimental variation).<br>4. The metabolic analysis was carried out using three biological repeats and in each case three technical repeats. We collected at least independent biological experiments for cell-based and in vitro biochemical studies, as is the common practice. Power calculations were used to assess the numbers of mice required for our studies and we used 9 mice in both the treated and control groups.<br>5. All western blots were carried out in triplicate and the data represent at least three biological replicates. This is standard within this field, and is a sufficient number of repeats to obtain statistically meaningful data.
Data exclusions	No data were excluded from this study
Replication	All experiments were replicated successfully. The N values for all experiments are shown in the figure legends. No data were excluded from the study.
Randomization	All samples were allocated to experimental groups randomly.
Blinding	Animal work: These experiments were blinded. Drug adminsitration and endpoint monitoring was performed by CRUK Beatson Institute Biological Services facility staff without knowledge of genotype.<br><br>Cell work: Blinding was not used for experimental cell biology. The correct compound will need to be used/administered in each case so blinding is not possible for data collection.<br><br>Tumour samples: Blinding was not possible for the analysis of the mesothelioma data samples compared to the control cells. The mesothelioma tumour cells have a very distinct growth characteristics and it is very clear which cells were tumour versus control.

Reporting for specific materials, systems and methods

We require information from authors about some types of materials, experimental systems and methods used in many studies. Here, indicate whether each material, system or method listed is relevant to your study. If you are not sure if a list item applies to your research, read the appropriate section before selecting a response.

Materials & experimental systems

n/a	Involved in the study
☐	☒ Antibodies
☐	☒ Eukaryotic cell lines
☒	☐ Palaeontology and archaeology
☐	☒ Animals and other organisms
☐	☒ Human research participants
☒	☐ Clinical data
☒	☐ Dual use research of concern

Methods

n/a	Involved in the study
☒	☐ ChIP-seq
☒	☐ Flow cytometry
☒	☐ MRI-based neuroimaging

Antibodies

Antibodies used

Primary antibodies. All used at 1:1000
4E-BP1 Cell Signalling Cat. #9644S
ATP5A antibody Abcam Cat. #ab14748
ATP5A Proteintech Cat. #14676-1-AP
ATP5F1 antibody Abcam Cat. #ab154564
ATPB abcam Cat. #ab14730
Beta-Actin Sigma Cat. #A1978
COX8A Abcam Cat. #ab191915
COX7B Abcam Cat. #ab14062
Cytokeratin cell signalling Cat. #8547
Dap5 Cell Signalling Cat. #5169
DDX3 Cell Signalling Cat. #2635
DRP1 (D6C7) Rabbit mAb Cell Signalling Cat. #8570S
eEF2 Abcam Cat. #ab75748
eIF4A1 cell signalling Cat. #2490
eIF4A2 cell signalling Cat. #2013
eIF4E Abcam ab33766
eIF4G1 Cell Signalling Cat. #C45A4
eIF6 Cell Signalling Cat. #3263
Ki67 SIGMA Cat. #SAB5700770
L15 Ribosomal Protein SIGMA Cat. #HPA044425
L24 Ribosomal Protein SIGMA Cat. #SAB2700500
L7a Ribosomal Protein Cell Signalling Cat. #2415
Mitofusin 2 antibody Abcam Cat. #ab56889
MTFP1 Polyclonal Antibody Thermo Fisher # PA5-50457
mTOR Cell Signalling 2983S
NDUFB8 Proteintech 14794-1-AP
NDUFV2 Rabbit Polyclonal Antibody Proteintech Cat. #15301-1-AP
NEO1 Abnova PAB22072
p70S6K Cell Signalling Cat. #9234
Phospho 4E-BP1 (Ser65) Cell Signalling Cat. #9456S
Phospho AKT (Ser473) cell signalling Cat. #4060
Phospho AKT (T308) cell signalling Cat. #13038
Phospho mTOR (Ser2448) Cell Signalling Cat. #2971S
Phospho p70S6K(T389) Thermo Fisher Cat. #MA5-15202,
Phospho-DRP1 (Ser616) (D9A1) Cat. #Cell Signalling 4494S
Phospho-S6 Ribosomal Protein (Ser235/236) Cat. #Cell Signalling
Phospho-S6 Ribosomal Protein (Ser240/244) Cell Signalling Cat. #2215S
phospho-eEF2 (T56). abcam Cat. #ab82981
Puromycin (12D10) MerkMillipore Cat. #MABE343
S15a Ribosomal Protein SIGMA Cat. #HPA047103
SDHB abcam Cat. #ab14714
S6 Ribosomal Protein Cell Signalling Cat. #2211
Total OXPHOS Human WB Antibody Cocktail abcam Cat. #ab110411
UQCRC2 Proteintech Cat. #14742-1-AP

Secondary Antibodies
HRP conjugated goat anti-rabbit: GE Healthcare Cat. #NA934V. Used at 1:10,000
Alexa Fluor 488 goat anti-mouse: life technologies Cat# A11001 Used at 1:2000
Alexa Fluor 488 goat anti-rabbit: life technologies Cat# A11008. Used at 1:1000
Alexa Fluor 568 goat anti-mouse: life technologies Cat# A11004. Used at 1:1000
Alexa Fluor 568 goat anti-rabbit: life technologies Cat# A11011. Used at 1:1000

Validation	Abcam<br>Antibodies were tested by immunoprecipitation of the relevant proteins from whole cell lysates and western blotting. A single band of the correct molecular weight as detected.<br>Phosphorylated antibodies were tested by pre-treating extracts with a phosphatase and then demonstrating that the phospho-specific band was no longer visible by western blot analysis<br><br>Cell signalling<br>Antibodies were tested by western blot analysis and by immunoprecipitation from cell lysates.<br>Phosphospecific antibodies were tested using cell stimulation which is known to cause large changes in phosphorylation. Insulin exposure was used for all proteins listed with the exception of phosphoDRP1 which used treatment with nocodozole which is known to change the phosphorylation status of this protein.<br><br>Proteintech<br>Antibodies were validated by testing against the purified protein and cell extracts run on the same gel. A single band was detected in each case.<br><br>Thermofisher<br><br>Antibodies were tested in whole cell extracts of serum-starved HeLa cells treated which were then treated with IGF1 to induce phosphorylation. Then western blotted was used. To confirm specificity, competition was performed by preincubation with the phosphopeptide to inhibit the antibody.<br><br>SIGMA<br>Western blots were carried out on total cell lysates and a single band of the correct size was tested in conjunction with the purified protein.<br><br>Merkmillipore<br>HEK293 lysates were made from cells treated with Puromycin or cycloheximide. The antibody only detected puromycin-incorporated neosynthesized proteins, with no reaction against the cycloheximide treated samples.

Eukaryotic cell lines

Policy information about cell lines

Cell line source(s)	These were generated from patients tumours see Chernova, T., et al. Molecular profiling reveals primary mesothelioma cell lines recapitulate human disease. Cell Death Differ 23, 1152-1164 (2016).
Authentication	cell lines are all authenticated as published in Chernova et al 2016. Moreover they are available from MesoBank UK and each line has a passport showing validation
Mycoplasma contamination	I confirm that all cells are regularly tested and are mycoplasma free
Commonly misidentified lines<br>(See ICLAC register)	None

Animals and other organisms

Policy information about studies involving animals; ARRIVE guidelines recommended for reporting animal research

Laboratory animals	mice C57BL6 Cdkn2a-/-;Nf2fl/fl;Tp53fl/fl were generously provided by Anton Berns, Netherlands Cancer Institute (NKI) and their induced phenotype was described previously<br>Tumour initiation was induced in young adult mice (aged 8-10 weeks) and mice were maintained until predefined humane clinical endpoints were reached. Both males and females were included on all treatment and control arms.<br>All experiments involving mice were approved by the local animal welfare (AWERB) committee and conducted under UK Home Office licence, PE47BC0BF (DJM, Glasgow) and were compliant with the ARRIVE guidelines (https://www.nc3rs.org.uk/arrive-guidelines). Mice were maintained on a constant 12hr light/dark cycle under controlled climate (19-22oC; 45-65% humidity), fed and watered ad libitum, and all were mixed background (FVBN and C57Bl/6). Mice bearing the combination of target alleles Cdkn2a-/-;Nf2fl/fl;Tp53fl/fl were generously provided by Anton Berns, Netherlands Cancer Institute (NKI) and their induced phenotype was described previously (47). Genotyping was performed by Transnetyx Inc.. Tumour initiation was induced in young adult mice (aged 8-10 weeks) and mice were maintained until predefined humane clinical endpoints were reached. Lentivirus expressing Cre recombinase was purchased from the University of Iowa vector core facility. Long fibre amosite asbestos was generously provided by Rodger Duffin (MRC Inflammation Unit, Edinburgh) and intrapleural injection of virus and asbestos were performed as previously described (17). AZ8186 and AZ2014 were supplied by Astra Zeneca under MTA/RCA with the CRUK Beatson Institute. Drugs were dissolved and administered in 0.5% HPMC, 0.1% Tween 20, H2O vehicle. Both males and females were included on all treatment and control arms.

Wild animals	none
Field-collected samples	None
Ethics oversight	All experiments involving mice were approved by the local animal welfare (AWERB) committee and conducted under UK Home Office licence, PE47BC0BF (DJM, Glasgow) and were compliant with the ARRIVE guidelines (https://www.nc3rs.org.uk/arrive-guidelines).

Note that full information on the approval of the study protocol must also be provided in the manuscript.

Human research participants

Policy information about studies involving human research participants

Population characteristics	The patient cohort represent a continuous cohort of surgical mesothelioma patients, and no further selection was performed. Recruitment was achieved by a simple retrospective search of the histopathology database, and all patients with sufficient archival tissue were included in the study. We have not identified the introduction of any significant biases
Recruitment	No biases were introduced. These were either formalin fixed tissue samples from patients who agreed to donate under the ethics described below or freshly resected samples also covered by the ethics below.
Ethics oversight	Human investigations were performed after Research Ethics Committee approval (LREC 08/H0406/226). All tissue and data used to facilitate the construction of the TMAs was collected under NHS ethics agreement 14/EM/1159 approved by the Northampton committee of the National 20

Note that full information on the approval of the study protocol must also be provided in the manuscript.
